# Comparison of skeletal and soft tissue pericytes identifies CXCR4^+^ bone forming mural cells in human tissues

**DOI:** 10.1038/s41413-020-0097-0

**Published:** 2020-05-22

**Authors:** Jiajia Xu, Dongqing Li, Ching-Yun Hsu, Ye Tian, Leititia Zhang, Yiyun Wang, Robert J. Tower, Leslie Chang, Carolyn A. Meyers, Yongxing Gao, Kristen Broderick, Carol Morris, Jody E. Hooper, Sridhar Nimmagadda, Bruno Péault, Aaron W. James

**Affiliations:** 1 Departments of Pathology, Johns Hopkins University, Baltimore, 21205 MD USA; 2 Department of Microbiology, Wuhan University School of Basic Medical of Science, Wuhan, Hubei P.R. China; 3 Department of Oral and Maxillofacial Surgery, School of Stomatology, China Medical University, Shenyang, Liaoning P.R. China; 4 Departments of Orthopaedics, Johns Hopkins University, Baltimore, 21205 MD USA; 5 Departments of Plastic Surgery, Johns Hopkins University, Baltimore, 21205 MD USA; 6 Departments of Autopsy Service, Johns Hopkins University, Baltimore, MD 21205 USA; 7 Departments of Radiology and Radiological Science, bon, Baltimore, 21205 MD USA; 8 UCLA and Orthopaedic Hospital Department of Orthopaedic Surgery and the Orthopaedic Hospital Research Center, Los Angeles, CA 90095 USA; 9 Center for Cardiovascular Science and MRC Center for Regenerative Medicine, University of Edinburgh, Edinburgh, UK

**Keywords:** Bone, Diseases

## Abstract

Human osteogenic progenitors are not precisely defined, being primarily studied as heterogeneous multipotent cell populations and termed mesenchymal stem cells (MSCs). Notably, select human pericytes can develop into bone-forming osteoblasts. Here, we sought to define the differentiation potential of CD146^+^ human pericytes from skeletal and soft tissue sources, with the underlying goal of defining cell surface markers that typify an osteoblastogenic pericyte. CD146^+^CD31^−^CD45^−^ pericytes were derived by fluorescence-activated cell sorting from human periosteum, adipose, or dermal tissue. Periosteal CD146^+^CD31^−^CD45^−^ cells retained canonical features of pericytes/MSC. Periosteal pericytes demonstrated a striking tendency to undergo osteoblastogenesis in vitro and skeletogenesis in vivo, while soft tissue pericytes did not readily. Transcriptome analysis revealed higher CXCR4 signaling among periosteal pericytes in comparison to their soft tissue counterparts, and CXCR4 chemical inhibition abrogated ectopic ossification by periosteal pericytes. Conversely, enrichment of CXCR4^+^ pericytes or stromal cells identified an osteoblastic/non-adipocytic precursor cell. In sum, human skeletal and soft tissue pericytes differ in their basal abilities to form bone. Diversity exists in soft tissue pericytes, however, and CXCR4^+^ pericytes represent an osteoblastogenic, non-adipocytic cell precursor. Indeed, enrichment for CXCR4-expressing stromal cells is a potential new tactic for skeletal tissue engineering.

## Introduction

The vasculature connects all human tissues. Beyond being a conduit for gas exchange, the vasculature houses mesenchymal progenitor cells that actively participate in tissue renewal and repair.^1,2^ A perivascular niche for mesenchymal stem/stromal cells (MSCs) has been observed across organ systems,^3–6^ and in human tissues microvascular CD146^+^ pericytes are best studied as a cellular forerunner of conventional, culture-derived MSCs.^7,8^

Despite the ubiquitous distribution of pericytes, their tissue-specific functions remain shrouded in mystery. Data to date support the notion that pericytes have organ-specific repair functions while maintaining MSC properties. For example, pericytes around articular cartilage are primed for chondrogenesis,^9^ while dental pulp pericytes spontaneously regenerate dentin.^10^ Similarly, brain-derived pericytes are primed to participate in neural repair.^11,12^ In a striking example of tissue specificity, pericytes within the renal stroma secrete bioactive renin to regulate blood volume.^13^ Importantly, these tissue-specific pericytes retain a canonical feature of all known pericytes—capability for multilineage differentiation.^14–16^ For example, and despite their heterologous origin, human adipose tissue-derived pericytes (and related perivascular cells) have been used extensively to regenerate bone by our research group across four preclinical models^3,17–23^ (see^24,25^ for reviews). Clearly some plasticity exists in the regenerative ability of human pericytes, yet identification of subsets of human pericytes with heterologous differentiation potential is essentially absent. In sum, understanding how pericytes regenerate their own tissue microenvironment, or conversely adopt a heterologous cellular fate, is of central importance for cell-based efforts in regenerative medicine.

Multiple data points suggest that skeleton associated pericytes, whether housed within periosteum or bone marrow, are important mediators of bone homeostasis and repair. Cell lineage tracing in avian chimaeras and reporter transgenic mice have shown that during embryonic endochondral ossification, a subset of osteoprogenitor cells marked in mice by Osx expression are carried from the surrounding limb mesenchyme, attached to the blood vessels that invade the cartilaginous anlagen of long bones.^8,9^ Before the advent of cell lineage tracing, the use of intravascular dyes that label mural cells suggested that periosteal pericytes participate in osteochondral repair.^26,27^ Later studies using smooth muscle actin (SMA) perivascular reporter animals^28^ or NG2 pericyte reporter mice^29^ also suggested that endogenous pericytic cells give rise to bone cells after fracture. Within the bone marrow, perivascular cells expressing nestin and leptin receptor can directly contribute to osteoblasts and chondrocytes.^30,31^ These aggregate studies suggest that endogenous pericytes play an important and direct role in skeletal development and repair.

Here, we employed a detailed examination of CD146^+^ human pericytes derived from either skeletal or soft tissue depots. Although multipotentiality was a conserved feature across all pericyte populations, striking regional specification existed in the tendency for pericytes to form osteoblasts. Periosteum-derived CD146^+^ pericytes demonstrated skeletogenic potential accompanied by an enrichment for CXCR4 signaling activity. Ectopic bone formation mediated by periosteal pericytes was dependent on intact CXCR4 signaling. Most strikingly, CXCR4^+^ human pericytes derived from soft tissue sources represent an osteoblast, rather than adipocyte precursor cell.

## Results

### Visualization, isolation, and characterization of human CD146^+^ periosteal pericytes

**Fig. 1:**
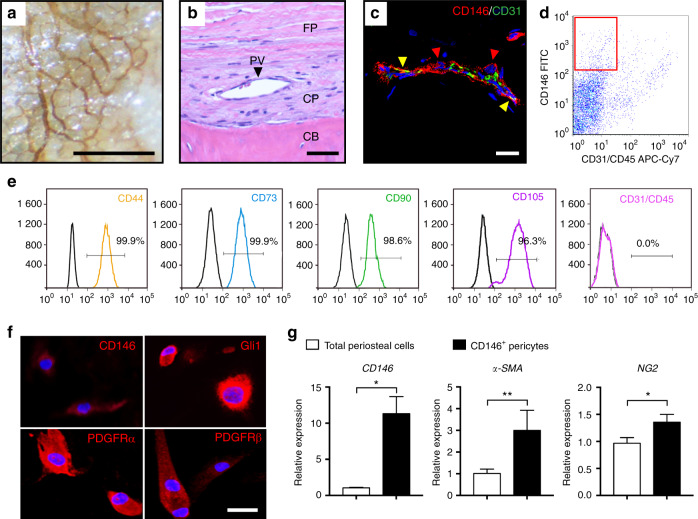
Periosteal CD146^+^ pericytes demonstrate progenitor cell features when purified by FACS. **a** CD146 whole mount immunohistochemical staining of human periosteum. Scale bar: 500 μm. **b** H&E appearance of human periosteum. CB cortical bone, CP cambial layer of periosteum, FP fibrous periosteum, PV periosteal vessel. Scale bar: 50 μm. **c** Immunofluorescent staining of periosteal blood vessels. Merged image, highlighting CD146^+^CD31^−^ pericyte (red arrowhead), and CD31^+^CD146^+^ endothelium (yellow arrowheads). Scale bar: 20 μm. **d** CD146^+^ pericyte isolation from human periosteum. Among the CD31^−^CD45^−^ non-endothelial/non-hematopoietic cell population, a CD146^+^ cell population (red box) is isolated. **e** Flow cytometry among freshly isolated CD146^+^ periosteal pericytes, including near universal expression of CD44, CD73, CD90, and CD105, and lack of CD31 and CD45 (shown together). Frequency of expression is shown in relation to isotype control antibody (colored versus black lines). **f** Immunofluorescent detection of pericyte and putative mesenchymal stem cell markers within purified CD146^+^ periosteal pericytes in culture, including CD146, Gli1, PDGFRα, and PDGFRβ. White scale bar: 50 μm. **g** Pericyte markers were detected by qRT-PCR between total periosteal cells and purified CD146^+^ periosteal pericytes from the same patient sample, including *CD146*, *α-SMA*, and *NG2*. *P < 0.05; **P < 0.01

Pericytes were first visualized within human periosteum as a CD146 (Mel-CAM) expressing perivascular cell population (Fig. 1). Whole mount immunohistochemistry on human uninjured periosteum demonstrated a delicate, anastomosing network of vasculature along the periosteal surface (Fig. 1a). Periosteal blood vessels were most frequent in the cambial rather than fibrous layer of the periosteum (Fig. 1b), and were highlighted by immunofluorescent immunohistochemical staining as abluminal CD146^+^CD31^−^ cells within the tunica intima, while CD31 marked endothelial cells (Fig. 1c). Human periosteal pericytes were isolated as the CD146^+^CD31^−^CD45^−^ fraction of the enzymatic digestion of human periosteum, using methods adapted from adipose tissue^3^ (Fig. 1d). Mean frequency of CD146^+^CD31^−^CD45^−^ pericytes within the periosteum was 4.87% (±3.09% SD) (Supplementary Table S1). After culture expansion, markers that typify cultured ‘MSCs’^32^ were examined by a combination of flow cytometry and immunocytochemistry among CD146^+^ periosteal pericytes. By flow cytometry, the vast majority of CD146^+^ periosteal pericytes co-expressed CD44, CD73, CD90, and CD105 (Fig. 1e, 96.3%–99.9% co-expression, passage 4 cells shown). As expected, no expression of CD31 or CD45 was seen among CD146^+^CD31^−^CD45^−^ pericytes (Fig. 1e). Immunocytochemistry showed continued expression of CD146 among culture-expanded CD146^+^ periosteal pericytes, as well as expression of putative pericyte or progenitor cell markers, including Gli1, PDGFRα (platelet-derived growth factor receptor α) and PDGFRβ (Fig. 1f, passage 4 shown). In comparison to unpurified periosteal cells derived from the same patient sample, CD146^+^ periosteal pericytes were enriched for gene transcripts of pericytes,^33,34^ including *CD146, αSMA*, and *NG2* (Fig. 1g, 10.74-, 2.95-, and 1.40-fold enrichment, respectively, in pericyte gene markers).

**Fig. 2:**
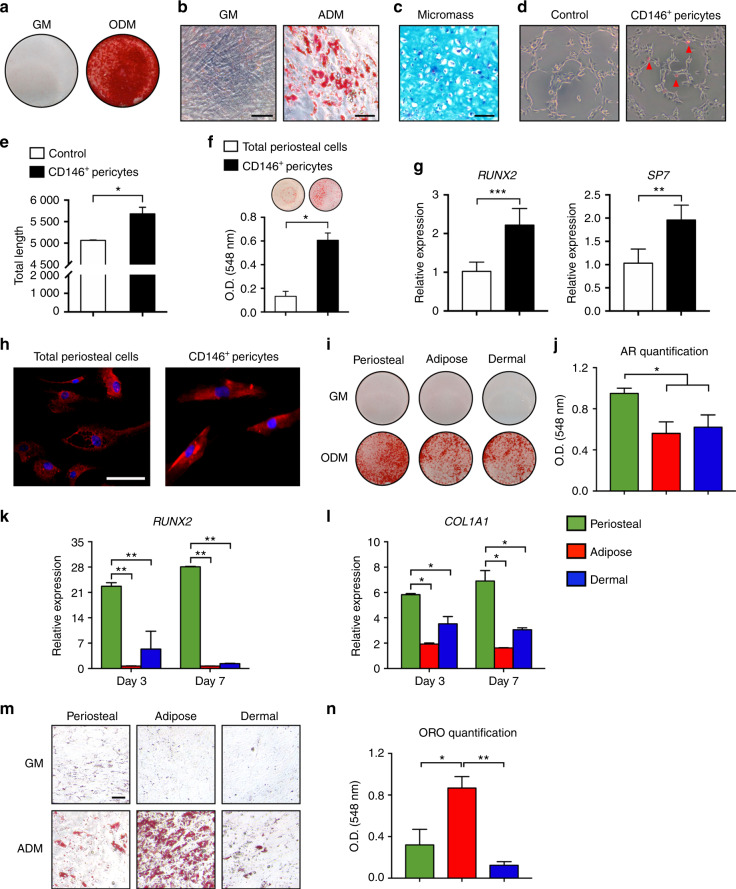
CD146^+^ pericytes show tissue-intrinsic programming for osteogenic and adipogenic differentiation. **a** Alizarin red S staining of bone nodules (day 7 of differentiation). **b** Oil red O staining of intracellular lipid droplets (day 10 of differentiation). Scale bar: 100 μm. **c** Alcian blue staining of CD146^+^ pericytes as a high-density micromass (day 21 of differentiation). Scale bar: 50 μm. **d**, **e** Tubule formation assay. HUVECs were seeded in culture alone, or in co-culture with purified periosteal CD146^+^ pericytes for 2 h. Before co-culture, CD146^+^ periosteal pericytes were labeled with red fluorescent dye (PKH26). **d** Representative images at ×10 magnification. **e** Quantification of tubule length. **f**–**h** Osteogenic differentiation of total unpurified periosteal cells and CD146^+^ periosteal pericytes derived from the same patient sample. **f** Alizarin red S staining at day 7 of bone nodules (above) and photometric quantification (below). **g** Osteogenic gene expression between total periosteal cells and purified CD146^+^ periosteal pericytes, including *runt-related transcription factor 2* (*RUNX2*) and *osterix* (*SP7*) at day 7 of differentiation. **h** Immunofluorescent staining for osteocalcin (OCN) in total periosteal cells and purified CD146^+^ periosteal pericytes. Scale bar: 50 μm. **i**–**l** Osteogenic differentiation of periosteal, adipose, and dermal CD146^+^ pericytes. **i** Alizarin red (AR) staining at day 7 of osteogenic differentiation among periosteal, adipose, and dermal CD146^+^ pericytes. **j** Photometric quantification of AR staining in **i**. **k**, **l** Osteogenic gene expression among periosteal, adipose, and dermal CD146^+^CD31^−^CD45^−^ pericytes, including (**k**) *RUNX2*, and (**l**) *Type I collagen* (*COL1A1*) at day 7 of differentiation. **m**, **n** Adipogenic differentiation of periosteal, adipose, and dermal CD146^+^ pericytes. **m** Oil red O (ORO) staining at day 10 of adipogenic differentiation. Black scale bar: 50 μm. **n** Photometric quantification of Oil red O staining in **m**. GM growth medium, ODM osteogenic differentiation medium, ADM adipogenic differentiation medium. *P < 0.05; **P < 0.01; ***P < 0.001

Canonical attributes of human pericytes were next assessed among CD146^+^ periosteal cells, including multipotent differentiation potential and paracrine-induced vascular tubulogenesis (Fig. 2a–h). In monolayer culture and with osteoinductive medium, confluent bone nodules were observed by Alizarin red (AR) staining (Fig. 2a). With adipogenic medium, Oil red O highlighted intracellular lipid droplets (Fig. 2b). In high-density micromass, a chondrocytic morphology was adopted, accompanied by cartilaginous matrix highlighted by Alcian blue staining (Fig. 2c). The tubulogenic attributes of CD146^+^ periosteal cells were next assessed by co-culture with human umbilical vein endothelial cells (HUVECs) (Fig. 2d, e). CD146^+^ periosteal pericytes were labeled with PKH lipophilic dye to permit visualization in co-culture. CD146^+^ periosteal pericytes adopted a perivascular location after co-culture (Fig. 2d), with increased tubule length in comparison to HUVEC cells alone (Fig. 2e). The osteogenic differentiation of CD146^+^ periosteal pericytes was notably swift (see again Fig. 2a), leading to further analysis. Here, the osteogenic differentiation of CD146^+^ periosteal pericytes was compared with that of unpurified periosteal stromal cells from the same patient sample (Fig. 2f–h, passage 3 examined for each cell population). CD146^+^ periosteal pericytes mineralized to a greater degree than unpurified stromal counterparts, as assessed by AR staining and photometric quantification (Fig. 2f). In support of this finding, gene expression confirmed a relative enrichment in the transcription factors *RUNX2* and *SP7* (*Osterix*) among differentiated CD146^+^ periosteal pericytes (Fig. 2g, 2.17- and 1.90-fold increase in transcripts among periosteal pericytes, respectively). Immunofluorescent staining for the terminal differentiation marker osteocalcin (OCN) was also more uniform and stronger among differentiated CD146^+^ periosteal pericytes (Fig. 2h). Thus, CD146^+^ periosteal pericytes represent a potent osteogenic population, but which also demonstrate canonical features of pericytes, including the capability of multilineage differentiation and paracrine-induced vascular tubulogenesis.

### Comparative differentiation and skeletogenic potential of human CD146^+^ pericytes from periosteal and soft tissue depots

The unexpectedly high mineralization level of CD146^+^ periosteal pericytes prompted a more thorough comparison to human pericytes from other tissue depots, including CD146^+^ pericytes derived from human adipose tissue and human dermis. The mean frequencies of FACS-purified CD146^+^ pericytes were similar across both soft tissue depots (Supplementary Table S1), with CD146^+^CD31^−^CD45^−^ pericytes representing 5.30% (±3.86% SD) of total cells from adipose tissue and 11.80% (±6.49% SD) from dermis. Representative FlowJo plots for isolation from adipose tissue of CD146^+^ pericytes, as well as their capacity for multilineage differentiation, are shown in Supplementary Fig. S1. The relative osteogenic differentiation potential among CD146^+^ pericytes across tissue depots was next assessed (Fig. 2i–l). Under osteogenic differentiation conditions, although all populations were able to form bone nodules, CD146^+^ periosteal pericytes formed significantly greater bone nodules as visualized by AR staining (Fig. 2i) and photometric quantification (Fig. 2j, 1.70- and 1.53-fold increase in bone nodules in periosteal as compared with adipose or dermis-derived cells). As well, a relative enrichment for gene transcripts associated with osteogenic differentiation was observed among CD146^+^ periosteal pericytes, including the master osteogenic transcription factor *RUNX2* (Fig. 2k, 4.24–40.69 fold increase among periosteal pericytes at days 3 and 7 of differentiation) as well as the matrix protein *type 1 collagen* (*COL1A1*) (Fig. 2l, 1.65–4.28-fold increase among periosteal pericytes at days 3 and 7 of differentiation). Converse experiments with CD146^+^ periosteal, adipose, and dermal pericytes were next performed under adipogenic differentiation conditions (Fig. 2m, n). Again, some degree of lipid accumulation was identified across all three tissue sources of CD146^+^ pericytes. Nevertheless, the degree to which cells underwent adipogenic differentiation was starkly dissimilar, with adipose tissue-derived CD146^+^ pericytes demonstrating substantially increased adipogenic differentiation by Oil red O staining (Fig. 2m) and photometric quantification (Fig. 2n, 2.71- and 6.96-fold increase among adipose-derived pericytes in comparison to periosteal- or dermal-derived cells). Thus, purified CD146^+^ pericytes retain tissue-intrinsic differences in relative osteogenic and adipogenic potentials, and tissue of origin corresponds to the degree of osteo- or adipocytic differentiation.

**Fig. 3:**
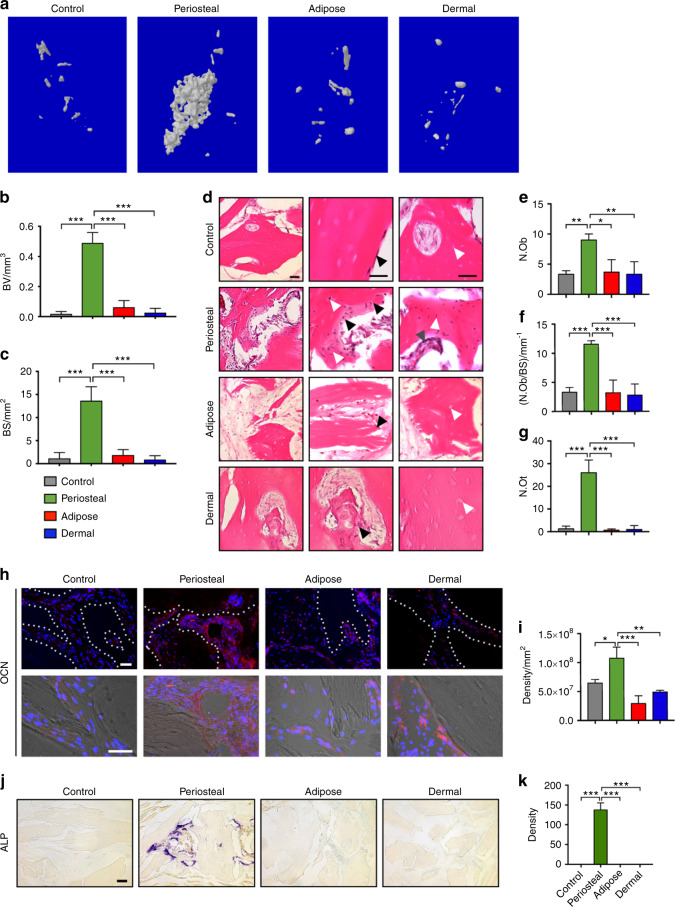
Relative skeletogenic potential of periosteal, adipose, and dermal CD146^+^ pericytes. CD146^+^CD31^−^CD45^−^ pericytes derived from periosteum, adipose, or dermis were implanted intramuscularly. Bone formation was assayed after four weeks. **a** Representative microcomputed tomography reconstruction images of the implant site among CD146^+^CD31^−^CD45^−^ pericytes derived from periosteum, adipose, or dermis. A demineralized bone matrix (DBM) putty was used as a scaffold carrier. **b** Mean bone volume (BV) among each treatment group. **c** Mean bone surface (BS). **d** Representative histologic appearance by routine H&E of pericyte implants, from periosteal, adipose, and dermal sources. Left-hand column shows low magnification image. Middle column highlights bone edges. Right-hand columns highlights osteocytic lacunae. Black arrowheads indicate bone-lining cells. White arrowheads indicate osteocyte lacunae. Black scale bar: 50 μm. **e**–**g** Bone histomorphometric measurements among each treatment group, including (**e**) osteoblast number (N.Ob), (**f**) osteoblast number per bone surface (N.Ob/BS), and (**g**) osteocyte number (N.Ot). **h** Representative osteocalcin (OCN) immunohistochemistry, appearing red, with DAPI nuclear counterstain, appearing blue. Low magnification with dotted white lines demarcating edges of bone. High magnification of interstices between bone, with DIC overlay. White scale bar: 50 μm. **i** Quantification of OCN activity among each treatment group per mm^2^. **j** Representative alkaline phosphatase (ALP) staining among each treatment group. Black scale bar: 100 μm. **k** Quantification of ALP staining. *P < 0.05; **P < 0.01; ***P < 0.001

The observed tissue-intrinsic differences in osteogenic potential of human pericytes were next assayed in vivo. For this purpose, CD146^+^ periosteal, adipose, or dermal pericytes were implanted using the thigh muscle pouch model in NOD/SCID mice. Adapted from our past methods,^3,21^ 1 million total CD146^+^ pericytes from each tissue depot were implanted using a demineralized bone matrix (DBM) carrier (MTF Biologics, Edison, New Jersey). An acellular DBM control was used as a further comparison. After four weeks, significant de novo bone formation was observed among CD146^+^ periosteal pericytes only, as observed by microcomputed tomography reconstructions of the implant site (Fig. 3a). Quantitative analysis confirmed a relative increase in parameters of bone formation within periosteal pericyte implants, including a 27.01-fold increase in bone volume (BV) (Fig. 3b, green versus gray bars), and 11.19-fold increase in bone surface (BS) in comparison to the acellular control (Fig. 3c, green versus gray bars). In stark contrast, no significant increase in bone formation was observed with either adipose or dermal pericytes in relation to acellular control (Fig. 3b, c, compare blue, red, and gray bars). Histologic analyses across implant sites confirmed these radiographic findings (Fig. 3d–i). Hematoxylin and eosin (H&E) staining demonstrated distinctive features of new bone formation among CD146^+^ periosteal pericyte implants (Fig. 3d), including woven bone matrix deposition with more prominent bone-lining osteoblasts (black arrowheads), numerous bone-entombed osteocytes (white arrowheads), and bone marrow recruitment (gray arrowheads). These features were minimal to absent among either adipose or dermal pericyte implant groups, which instead had similar features to acellular control such as DBM carrier embedded in a fibrous matrix, with minimal bone-lining cells (black arrowheads), empty osteocyte lacunae of the DBM (white arrowheads), and no discernable evidence of bone marrow recruitment. Quantitative bone histomorphometric analysis confirmed these findings, demonstrating a significant increase in osteoblast numbers among periosteal pericyte implants (Fig. 3e, f), including an 2.7-fold increase in osteoblast number (Ob.N, Fig. 3e) and 3.51-fold increase in osteoblast number when normalized to BS (Ob.N/BS, Fig. 3f). Likewise, osteocyte numbers (N.Ot) were significantly increased among periosteal pericyte implants only (Fig. 3g, 19.5-fold increase in N.Ot in comparison to acellular control). Immunofluorescent detection of OCN demonstrated robust immunoreactivity among CD146^+^ periosteal pericyte implants, with less conspicuous staining across soft tissue pericytes or acellular control (Fig. 3h, appearing red). Semi-quantitative analysis of OCN immunoreactivity confirmed a relative enrichment among periosteal pericyte implants (Fig. 3i, 1.66-fold increase in comparison to acellular control). Staining for alkaline phosphatase (ALP) enzymatic activity again showed clear differences across pericyte sources, with abundant ALP activity among periosteal pericyte implants (Fig. 3j, appearing purple), confirmed by semi-quantitative histomorphometric analysis (Fig. 3k). Thus, human CD146^+^ periosteal pericytes represent a comparatively osteoblastogenic/skeletogenic cell population in comparison to soft tissue pericytes.

### Transcriptomic comparison of human CD146^+^ pericytes from periosteal and soft tissue depots

**Fig. 4:**
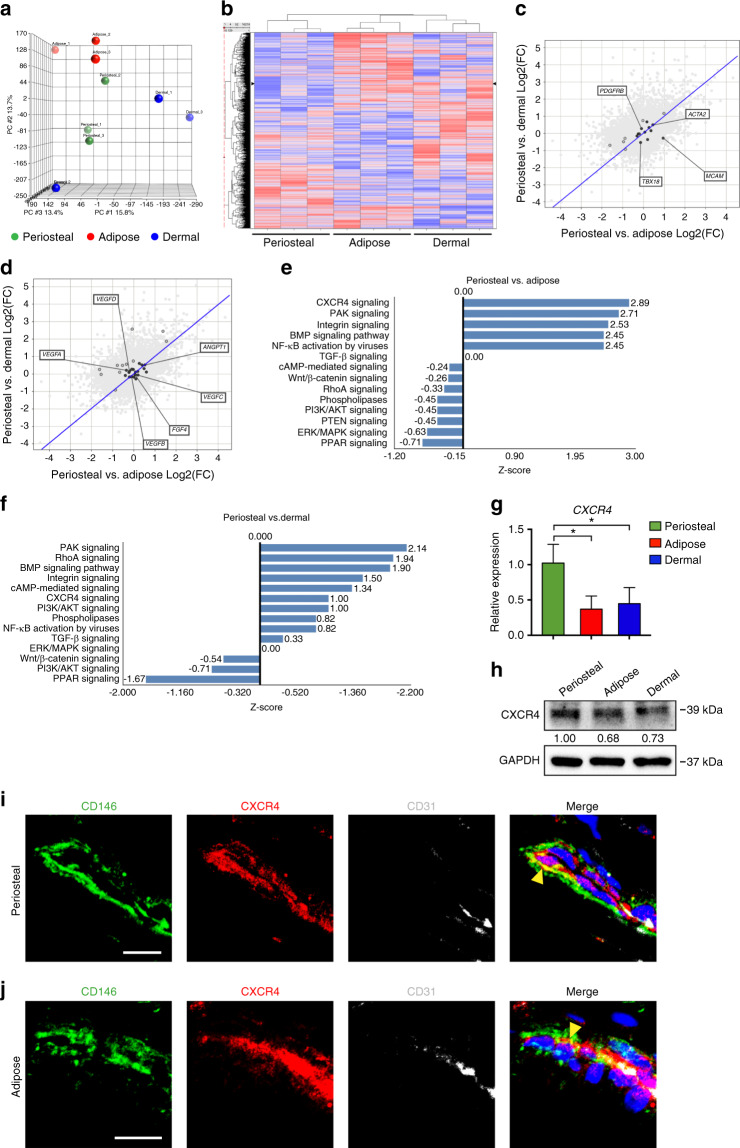
Transcriptome of periosteal, adipose, and dermal CD146^+^ pericytes. **a**–**f** Transcriptome of periosteal, adipose, and dermal CD146^+^ pericytes was analyzed by Clariom D microarray among undifferentiated, culture-expanded cells of equal passage. **a** Principal component analysis among periosteal, adipose, and dermal CD146^+^ pericytes. **b** Unsupervised hierarchical clustering among periosteal, adipose, and dermal CD146^+^ pericytes. **c** Expression of canonical pericyte markers among periosteal, adipose and dermal CD146^+^ pericytes, shown in quantile–quantile (Q–Q) plot. See Supplementary Table S5 for a complete list. **d** Expression of common angiogenic cytokines among periosteal, adipose, and dermal CD146^+^ pericytes, shown in Q–Q plot. See Supplementary Table S6 for a complete list. **e** Ingenuity Pathway Analysis (IPA) identified representative pathways that were upregulated (*Z*-score > 0) or downregulated (*Z*-score < 0) among periosteal versus adipose CD146^+^ pericytes. **f** IPA identified representative pathways that were upregulated (*Z*-score > 0) or downregulated (*Z*-score < 0) among periosteal versus dermal CD146^+^ pericytes. **g**, **h** Representative qRT-PCR (**g**) and Western blotting (**h**) analyses of CXCR4 expression in CD146^+^ periosteal, adipose, and dermal pericytes. **i** Immunofluorescent staining of periosteal tissue microvessels for CD146 (green), CXCR4 (red), and CD31 (white). Some periosteal vessels demonstrated CXCR4^+^CD146^+^CD31^−^ perivascular cells (yellow arrowhead). White scale bar: 10 μm. **j** Immunofluorescent staining of adipose tissue microvessels for CD146 (green), CXCR4 (red), and CD31 (white). Some adipose tissue vessels demonstrated CXCR4^+^CD146^+^CD31^−^ perivascular cells (yellow arrowhead). White scale bar: 10 μm. *P < 0.05

Tissue-intrinsic differences in pericyte differentiation potential were next investigated using transcriptomic analysis of three separate preparations of periosteal, adipose, and dermal CD146^+^ cells. Affymetrix Clariom D microarray analysis was performed to compare mRNA expression across pericytes from different tissue samples (Fig. 4a–h). Briefly, raw data, as.CEL files, were imported and quantile normalized into log notation following the RMA protocol using the Partek Genomics Suite v7.0 platform (Partek Inc., St. Louis, MO, USA). Two-tailed one-way ANOVA, Student’s *t* test was performed between the samples’ tissue type classes to determine which genes were differentially expressed. Transcripts were normalized by fragments per kilobasepair per million mapped (FPKM), and those with Log2 FPKM >−0.8 underwent further analysis. Among these, 28 932 annotated genes were expressed in all samples with 135 750 total RNA transcripts (21.3% of total, including 20 055 protein coding RNAs; 5 552 noncoding RNAs; and 2 813 pseudo RNAs per NCBI annotation). Clear separation between gene expression profiles was observed when comparing periosteal, adipose, and dermal pericytes, as revealed by principal component analysis (Fig. 4a) and unsupervised hierarchical clustering (Fig. 4b). Putative gene markers of human pericytes were cross-referenced within each tissue of origin (Fig. 4c, Supplementary Table S5). As visualized using Q–Q plots, pericyte markers^35^ such as actin alpha 2, smooth muscle (*ACTA2*), platelet-derived growth factor receptor beta (*PDGFRB*), and melanoma cell adhesion molecule (*MCAM*, CD146) were commonly expressed across cell preparations. Gene markers associated with the regulation of vasculogenesis were also commonly expressed among all cell preparations, such as angiopoietin (*ANGPT*), fibroblast growth factor 4 (*FGF4*), and vascular endothelial growth factor A (*VEGFA*) (Fig. 4d, Supplementary Table S6, GO term: angiogenesis).

QIAGEN Ingenuity Pathway Analysis (IPA) showed that many of the activated pathways among periosteal pericytes were associated with the positive regulation of osteogenesis, including for example CXCR4 signaling,^36^ integrin signaling,^37^ and BMP signaling^38^ (Fig. 4e, f, Supplementary Tables S7 and S9, *Z-*scores ranging from 1.0 to 2.887 when comparing periosteal to adipose or dermal pericytes). Conversely, downregulated signaling pathways among periosteal pericytes included those known to positively regulate adipogenesis, such as LXR/RXR activation,^39^ PPAR signaling,^40^ STAT3 pathway,^41^ and insulin receptor signaling^42^ (Fig. 4e, f, Supplementary Tables S8 and S10, *Z-*scores ranging from −2.309 to −0.707 when comparing periosteal to adipose or dermal pericytes). In sum, signaling pathway analysis across pericyte depots confirmed the importance of the tissue of origin in conferring the ability of pericytes to give rise to either osteoblasts or adipocytes in culture.

Among bone anabolic pathways enriched in periosteal pericytes, CXCL12–CXCR4 signaling was further pursued. We observed increased CXCR4 expression in periosteal pericytes as compared with other tissue depots, as shown by quantitative PCR and western blotting (Fig. 4g, h). Differential gene expression of *CXCL12* and its alternate receptor *ACKR3* were also observed by qPCR (Supplementary Fig. S2). Immunofluorescent staining for CXCR4 confirmed the presence of CXCR4^+^ cells within a pericytic location in many microvessels (Fig. 4i, j). Here, pericytic CXCR4 immunoreactive cells were seen in both periosteum (Fig. 4i) and adipose tissue (Fig. 4j) associated vessels. To further examine CXCR4 signaling among adipose pericytes, we reanalyzed an existing single-cell RNA sequencing library derived from a single human donor’s adipose tissue.^43^ Supervised clustering of gene expression profiles identified four cell types as previously described (Supplementary Fig. S3a).^43^ These included a ‘pericyte/smooth muscle cell (SMC)’ population that expressed *MCAM*, *ACTA2*, and *PDGFRB* (cluster 4) (Supplementary Fig. S3b**)**. Other groups included the previously termed ‘interstitial progenitors,’ expressing dipeptidyl peptidase-4 *(DPP4*) and *CD55* (cluster 1), ‘committed preadipocytes’ expressing dipeptidase 1 (*DEPP1*) and vascular adhesion molecule 1 (*VCAM1*) (cluster 2), and endothelial cells expressing endoglin (*ENG*) and platelet endothelial cell adhesion molecule 1 (*PECAM1*) (cluster 3). CXCR4 signaling genes as generated by IPA software were queried across these four human adipose cell populations (Supplementary Fig. S3c). Pericytes/SMCs in cluster 4 showed most abundant expression of genes involved in the CXCR4 signaling pathway, including *CXCR4* itself. In sum, CXCR4-expressing pericytes are present across skeletal and soft tissues. However, CXCR4 expression and CXCR4 signaling as predicated by pathway analyses are over-represented among periosteal pericytes.

### CXCR4 inhibition abrogates ectopic bone formation among CD146^+^ periosteal pericytes

**Fig. 5:**
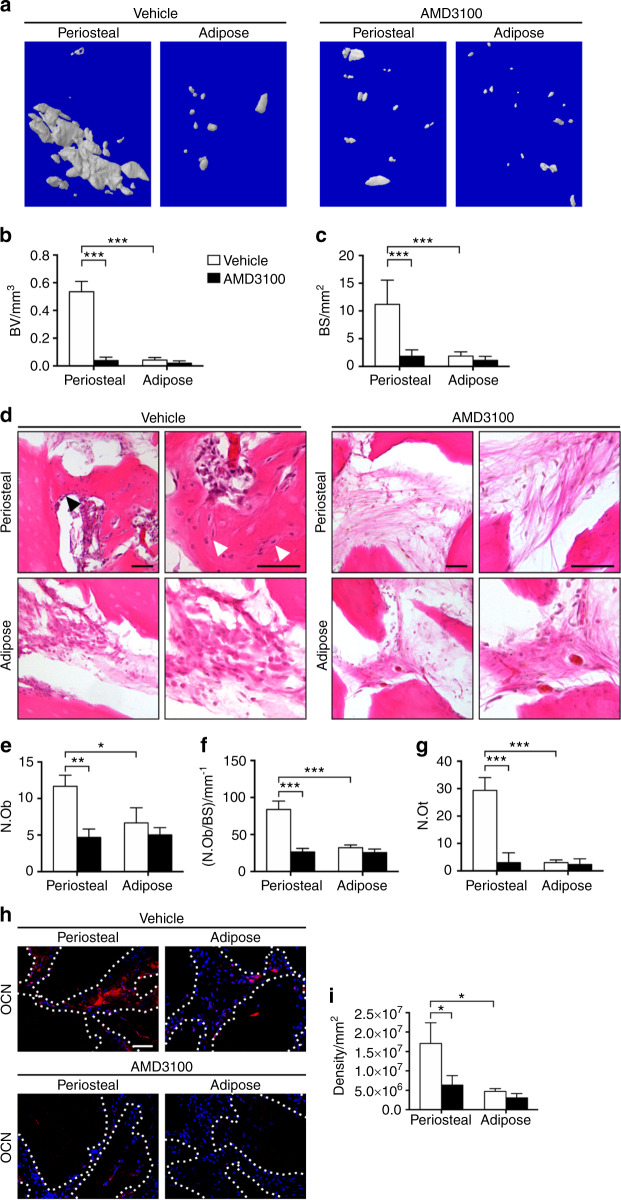
Inhibition of CXCR4 signaling impairs bone formation of implanted CD146^+^ pericytes. CD146^+^CD31^−^CD45^−^ pericytes derived from periosteum or adipose tissue were implanted intramuscularly with or without AMD3100 treatment. Bone formation was assayed after four weeks. **a** Representative microcomputed tomography reconstruction images of the implant site among CD146^+^ pericytes derived from periosteum or adipose with AMD3100 or vehicle control. A DBM was used as a scaffold carrier. **b** Mean bone volume (BV) among each treatment group. **c** Mean bone surface (BS). **d** Representative histologic appearance by routine H&E of periosteal or adipose tissue CD146^+^ pericyte implants, among host animals treated with AMD3100 or vehicle control. Each image shown at low and high magnification. Black scale bar: 50 μm. **e**–**g** Bone histomorphometric measurements among each treatment group, including (**e**) osteoblast number (N.Ob), (**f**) osteoblast number per bone surface (N.Ob/BS), and (**g**) osteocyte number (N.Ot). **h** Osteocalcin (OCN) immunohistochemistry, appearing red, with DAPI nuclear counterstain, appearing blue. Dotted white lines demarcated edges of bone. White scale bar: 50 μm. **i** Quantification of OCN activity in the implants was shown. *P < 0.05; **P < 0.01; ***P < 0.001

To test the association between CXCR4 signaling and pericyte-mediated osteogenic differentiation, we treated periosteal pericytes with the CXCR4 antagonist AMD3100. CXCR4 inhibition via AMD3100 inhibited osteogenic differentiation of periosteal pericytes in vitro, as shown by ALP staining and quantification (Supplementary Fig. S4a, b). Next, we returned to our pericyte intramuscular implantation model, now with systemic treatment with or without AMD3100 continuously over the assay duration. Here, periosteal or adipose tissue-derived CD146^+^ pericytes were again implanted in equal numbers (1 million cells/implants). AMD3100 or vehicle control was delivered daily (5 mg·kg^−1^), and bone formation was assessed four weeks thereafter. Under control conditions, periosteum-derived implants again demonstrated clearly increased radiographic evidence of bone formation, observed by microCT reconstructions of the implant site (Fig. 5a). Conversely, AMD3100 treatment abrogated ossification among periosteal pericyte implants, observed by qualitative (Fig. 5a) and quantitative microCT assessments (Fig. 5b, c, 92.85% reduction in BV and 83.64% reduction in BS among periosteal pericyte implants with AMD3100 treatment). Histologic analysis confirmed baseline histologic differences between periosteal and adipose tissue pericyte implants, including significant new bone formation, increased osteoblasts and osteocytes, and presence of bone marrow among periosteal pericyte implants (Fig. 5d, upper left). These features were again inconspicuous among adipose tissue-derived pericyte implants under control conditions (Fig. 5d, lower left). In marked contrast, periosteal pericyte implants among AMD3100 treated animals lacked these features, and instead showed DBM chips with inconspicuous bone-lining cells within predominantly fibrous tissue (Fig. 5d, upper right). Histomorphometric assessments demonstrated a significant reduction in osteoblast numbers (Fig. 5e, N.Ob), osteoblast number per BS (Fig. 5f, N.Ob/BS), and osteocyte numbers (Fig. 5g, N.Ot) among AMD3100 treated animals as compared with control-treated animals. Immunohistochemistry for OCN again demonstrated heightened antigen detection among periosteal pericyte implant sites, which was significantly reduced among AMD3100 treated implant sites (Fig. 5h, i). In sum, CXCR4 inhibition completely abrogated ectopic bone formation by CD146^+^ periosteal pericytes.

### CXCR4^+^ pericytes within human adipose tissue are osteoblast but not adipocyte precursors

**Fig. 6:**
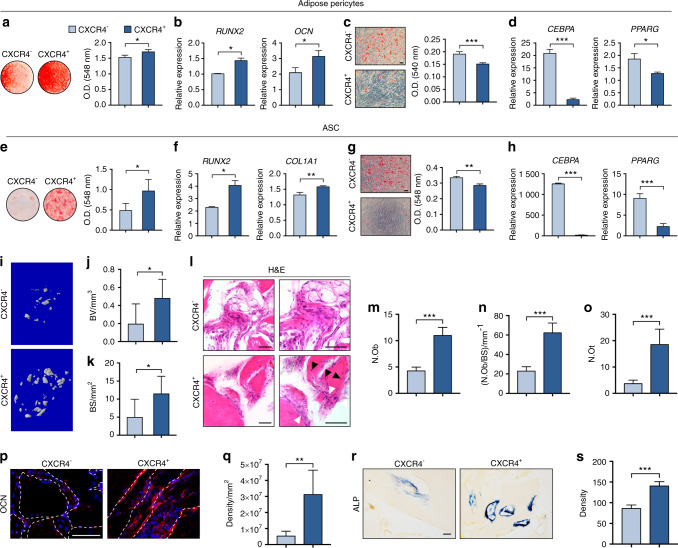
CXCR4-expressing adipose tissue pericytes are an osteoblastic/non-adipocytic progenitor cell. **a**, **b** Osteogenic differentiation of adipose tissue CXCR4^−^ or CXCR4^+^ pericytes, as assessed by Alizarin Red (AR) staining (**a**) and osteogenic gene expression (**b**), including *RUNX2* and *OCN* at day 7 of differentiation. **c**, **d** Adipogenic differentiation of adipose tissue CXCR4^−^ or CXCR4^+^ pericytes, as assessed by Oil red O (ORO) staining (**c**) at day 10 of differentiation and adipogenic gene expression (**d**), including *CEBPA* and *PPARG* at day 7 of differentiation. Black scale bar: 50 μm. **e**–**h** Unpurified adipose tissue stromal cells (ASCs) were purified into CXCR4^−^ and CXCR4^+^ cell populations, followed by osteogenic and adipogenic differentiation assays. **e** AR staining and photometric quantification at day 7 of differentiation. **f** Osteogenic gene expression, including *RUNX2* and *COL1A1* at day 7 of differentiation. **g** ORO staining and photometric quantification at day 10 of differentiation. Black scale bar: 50 μm. **h** Adipogenic gene expression, including *CEBPA* and *PPARG* at day 7 of differentiation. **i**–**k** CXCR4^−^CD31^−^CD45^−^ and CXCR4^+^CD31^−^CD45^−^ ASCs were implanted intramuscularly using a DBM carrier. **i** Representative microCT reconstruction images. **j** Bone volume (BV) and **k** bone surface (BS). **l** Representative histologic appearance by H&E of CXCR4^−^ or CXCR4^+^ ASC implants. Black scale bar: 50 μm. **m**–**o** Bone histomorphometric measurements, including (**m**) osteoblast number (N.Ob), (**n**) osteoblast number per bone surface (N.Ob/BS), and (**o**) osteocyte number (N.Ot). **p** Osteocalcin (OCN) immunohistochemistry, appearing red, with DAPI nuclear counterstain appearing blue. Dotted white lines demarcated edges of bone. White scale bar: 50 μm. **q** Quantification of OCN immunostaining. **r** Representative alkaline phosphatase staining, and **s** quantification. Black scale bar: 50 μm. *P < 0.05; **P < 0.01; ***P < 0.001

Having observed that a portion of human pericytes express CXCR4 in situ, we next attempted to determine if membranous CXCR4 expression highlights pericyte subpopulations with functional relevance. Among adipose-derived CD146^+^ pericytes, cells were further divided based on cell surface CXCR4 expression into CXCR4^+^ and CXCR4^−^ populations (Supplementary Fig. S5a, b, cell permeabilization revealed that essentially all cells expressed intracellular CXCR4). Osteogenic differentiation among CXCR4^+^ and CXCR4^−^ pericytes was first assessed (Fig. 6a, b). Although all pericyte subpopulations had basal ability to undergo osteogenic differentiation, CXCR4^+^ pericytes demonstrated higher mineralization rates (Fig. 6a), as well as increased transcripts for osteogenic markers (Fig. 6b). Converse experiments with CXCR4^+^ and CXCR4^−^ pericytes were next performed under adipogenic differentiation conditions (Fig. 6c, d). Marked reductions in lipid accumulation (Fig. 6c, 25.0% reduction) and transcripts for adipocyte markers were observed among CXCR4^+^ pericytes (Fig. 6d, 89.3% and 31.2% reduction in *CEBPA* and *PPARG* expression among CXCR4^+^ pericytes, respectively). Heightened osteoblastic potential among CXCR4^+^ rather than CXCR4^−^ pericytes was further confirmed among analogous experiments performed in pericytes of dermal origin (Supplementary Fig. S5c, d, 4.99-fold increase observed in mineralization among CXCR4^+^ dermal pericytes). Inconspicuous adipogenic differentiation was observed among either CXCR4^+^/^−^ dermal pericytes (Supplementary Fig. S5e, f). The osteoblastic/non-adipocytic phenotype of CXCR4^+^ pericytes was further validated using unsorted adipose stromal cell (ASC) preparations (Fig. 6e–h). First, osteogenic differentiation was impaired by the CXCR4 antagonist AMD3100 in unpurified ASCs (Supplementary Fig. S4c, d). Total unpurified ASCs defined by culture adherence were next FACS sorted by CXCR4 expression among total CD31^−^CD45^−^ cells (Supplementary Fig. S5g). Comparative osteogenic differentiation (Fig. 6e, f) and adipogenic differentiation (Fig. 6g, h) among CXCR4^−^ and CXCR4^+^ ASCs confirmed our findings within adipose tissue pericytes. By all markers, CXCR4^+^ ASCs represent an osteoblast/non-adipocyte precursor cell population. Additional phenotypic differences were observed between CXCR4^−^ and CXCR4^+^ ASCs. CXCR4^+^ cells showed significantly higher migratory potential in transwell migration assays (Supplementary Fig. S5h). Moreover, CXCL12 treatment induced migration in CXCR4^+^ but not CXCR4^−^ ASCs (Supplementary Fig. S5h).

Finally, we returned to intramuscular implantation to determine the skeletogenic potential of CXCR4^+^ versus CXCR4^−^ ASCs (Fig. 6i–s). A significant increase in ectopic bone was observed among CXCR4^+^ ASC implants in comparison to CXCR4^−^ ASCs derived from the same patient sample as shown by microCT imaging and quantitative analysis (Fig. 6i–k, 147.5% and 131.5% increase in BV and BS among CXCR4^+^ implants). CXCR4^+^ cell implant sites demonstrated an increase in woven bone formation (Fig. 6l), increase in osteoblast and osteocyte numbers (Fig. 6m–o), and enrichment for the osteogenic markers OCN and ALP (Fig. 6p–s). Thus, membranous CXCR4 expression within either soft tissue pericytes or adipose tissue total stroma identifies an osteoblast/non-adipocyte precursor cell.

## Discussion

In summary, although CD146^+^ human pericytes from diverse organs meet current definitions of stem/progenitor cells, pericytes also display profound tissue-intrinsic properties in bone- and fat-forming potential. This reinforces the tissue-specific properties of human pericytes observed by other research groups: for example, muscle-associated human pericytes were more myogenic in vitro and in vivo.^18^ Yet, functionally relevant intra-tissue diversity exists within human pericytes. As a newly distinguishing factor, membranous CXCR4 expression divides bone- from fat-forming human pericytes.

The ability of cells from adipose tissue to undergo heterologous differentiation into bone is well described, either using unfractionated stromal cells,^44^ or purified cell populations with a perivascular residence (see^25^ for a review). Rare genetic syndromes in human patients also demonstrate heterologous differentiation of fat resident cells into bone, where *GNAS* gene mutations in progressive osseous heteroplasia lead to bone formation particularly within the subcutaneous adipose niche^45^ (OMIM: 166350). Yet, more wide scale tissue engineering approaches for the transdifferentiation of adipose resident cells into bone cells have met with inconsistent results.^46^ The tissue-intrinsic diversity within human adipose stroma is likely a central contributing factor to the lack of success with this ‘fat-to-bone’ tissue engineering approach, and single-cell diversity within adipose tissue has recently been more apparent.^43^ Interestingly, using recent single-cell sequencing data,^43^ *CXCR4* transcripts and gene clusters of the CXCR4 signaling pathway preferentially associate to pericyte and SMCs within adipose tissue. Moreover, when these publicly available data sets are reanalyzed, newly described adipose tissue subpopulations such as DPP4^+^ ‘interstitial progenitors’ and ICAM1^+^ ‘committed preadipocytes’ clearly segregate based on CXCR4 signaling activity. It should be noted, however, that purified CD146^+^ human pericytes also co-express markers such as *DPP4, PREF1*, *ICAM2*, and *PPARG*, and it is not currently clear how human pericytes fit into the recently proposed developmental hierarchy of adipose tissue.^43^

In addition, the identification of a CXCR4-expressing pericyte population with increased ability to form bone tissue begs questions regarding the static versus migratory nature of endogenous human pericytes. When placed in contact co-culture, pericytes traffic across endothelial networks.^47^ The existence of migratory subsets of osteoprogenitor cells has been observed via classic parabiosis experiments,^48^ or infusion studies with culture-derived MSCs.^49–51^ In fact, the adhesion factor CD146 is well known to be aberrantly expressed across a diverse set of cell types when cells take up a subendothelial position.^52,53^ The possibility that CXCR4-expressing pericytes represent a migratory or trafficking cell, rather than a tissue-specific cell, is of ongoing investigation.

Several caveats exist toward the broader extrapolation of these results. First, despite the many studies examining the stem cell characteristics of pericytes,^54–58^ the MSC characteristics in vivo of at least a subpopulation of pericytes has been recently called into some question by a recent study using a Tbx18 transgenic reporter mouse.^47^ Tbx18 is a transcription factor selectively expressed in pericytes, smooth muscle, and other interstitial cell types. Despite multilineage differentiation potential in vitro, Tbx18^+^ pericytes did not display tissue-specific progenitor cell attributes in vivo.^47^ Although unresolved how much Tbx18 marks all pericytes or rather a subset of committed ones (some mouse organs are devoid of Tbx18^+^ pericytes),^47^ these data suggest that not all mural cells possess regenerative properties. Of note and in our study, *TBX18* transcripts were expressed across all human pericyte depots, albeit to a lesser degree in periosteal pericytes. Second, the extent to which hematopoietic stem cell support is a conserved versus tissue-restricted function of pericytes is controversial. For example, our own observations have shown that HSC support is a ubiquitous rather than tissue-restricted feature of CD146^+^ human pericytes.^59^ This lies at odds with observations by Sachetti et al. who found that bone marrow recruitment was an isolated feature of marrow-derived pericytes.^18^

In summary, skeletal and soft tissue pericytes differ in their relative differentiation potentials and basal abilities to form bone. Diversity exists in soft tissue pericytes, however, and CXCR4^+^ pericytes represent an osteoblastogenic, non-adipocytic cell precursor. Indeed, enrichment for CXCR4-expressing stromal cells is a potential new tactic for skeletal tissue engineering. For the future, detailed studies that improve upon our correlation between vessel of origin, distinguishing cell surface markers, and functional outcome posttransplantation are required to better understand the cellular identity and regenerative properties of human pericytes.

## Methods

### Isolation of periosteal, adipose, and dermal CD146^+^ pericytes

Unless otherwise stated, materials were purchased from Sigma-Aldrich (St. Louis, MO). CD146^+^ periosteal cells were isolated from human periosteum via fluorescence-activated cell sorting (FACS). Human periosteal tissue was obtained from adult patient donors under IRB approval at JHU with a waiver of informed consent, and was stored for <48 h at 4 °C before processing. Samples are summarized in Supplementary Table S1. The total periosteal cells of human periosteum were obtained by collagenase II digestion. Briefly, periosteum tissue was removed from bone, finely minced, and digested with a 1 mg·mL^−1^ type II collagenase in Dulbecco’s modified Eagle’s medium (DMEM) for 70 min under agitation at 37 °C. Cells were separated and removed by centrifugation. The cell pellet was resuspended in red blood cell lysis buffer (155 mmol·L^−1^ NH_4_Cl, 10 mmol·L^−1^ KHCO_3_, and 0.1 mmol·L^−1^ EDTA) and incubated at 37 °C for 5 min. After centrifugation, cells were resuspended in PBS and filtered at 100, 70, and 40 μm in turn. The resulting periosteal cells was further processed for cell sorting, using a mixture of the following directly conjugated antibodies: anti-CD31-APC-cy7 (1:100; BD Pharmingen, San Diego, CA), anti-CD45-APC-cy7 (1:30; BD Pharmingen), and anti-CD146-fluorescein isothiocyanate (1:100; Bio-Rad, Hercules, CA) (summary of antibodies presented in Supplementary Table S2). All incubations were performed at 4 °C for 15 min. The solution was then passed through a 70 μm filter and then run on a DakoCytomation MoFlo (Beckman, Indianapolis, IN, USA). FlowJo software was used for the analysis of flow cytometry data. In this manner, periosteal pericytes (CD146^+^CD31^−^CD45^−^) were isolated. In select studies, unsorted (unpurified) periosteal cells from the same patient samples were also isolated for culture.

The isolation of dermal and adipose pericytes was performed using an approach analogous to that of periosteal cell isolation, and has been previously published.^60^ Dermal pericytes were derived from microdissected dermis from elective abdominoplasty specimens from *n* = 3 adult, female donors. Adipose pericytes were derived from elective liposuction specimens from *n* = 3 adult, female donors. A similar staining and gating procedure was performed, with the addition of anti-CD34-APC (1:100, BD Pharmingen) to derive a CD146^+^CD31^−^CD45^−^CD34^−^ pericyte population. In select studies, pericytes or ASCs were further partitioned using anti-CXCR4-APC (1:5, BD Pharmingen).

FACS-purified CD146^+^ pericytes were either analyzed by flow cytometry, snap frozen for RNA isolation, applied in a mouse intramuscular implantation model, or culture expanded for in vitro studies. For in vitro expansion, all cells were cultured at 37 °C in a humidified atmosphere containing 95% air and 5% CO_2_. All cells were cultured in DMEM medium with 10% fetal bovine serum (FBS) (Gibco, Grand island, NY, USA) and 1% penicillin/streptomycin (Life technologies corporation, Gaithersburg, MD, USA). Medium was changed every 3 days unless otherwise noted.

### Flow cytometry

Cell surface markers were detected by flow cytometry after sorted CD146^+^ cells were expanded (passage 3). Unless otherwise specified, all antibodies were from BD Pharmingen (Supplementary Table S2). Briefly, cells were stained at a density of 1 × 10^5^ cells per mL in PBS with the following antibodies and dilutions: mouse anti human mAb anti-CD31-Apc-cy7 (1:30), anti-CD44-Alexa Fluor 700 (1:20), anti-CD45-Apc-cy7 (1:30), anti-CD73-PE (1:30), anti-CD90-FITC (1:30), and anti-CD105-PE-CF594 (1:20) were added separately and incubated at 4 °C for 15 min. Cells were then passed through a 70 μm filter and examined with a DakoCytomation MoFlo (Beckman). FlowJo software was used for the analysis of flow cytometry data.

### Osteogenic differentiation assays

CD146^+^ periosteal, adipose, and dermal pericytes or subpopulations based on CXCR4 expression of equal passage number were seeded in 24- or 48-well plates at a density of 2 × 10^5^ cells per mL. Osteogenic differentiation medium (ODM) consisted of DMEM, 10% FBS, 1% penicillin/streptomycin with 10 mmol·L^−1^ β-glycerophosphate, 50 μmol·L^−1^ ascorbic acid, and 1 mmol·L^−1^ dexamethasone. In total, 24 h after cell seeding, basal medium was replaced with ODM, replenished every 3 days. For AR staining, cells were washed with PBS and fixed with 4% paraformaldehyde at 7 days of differentiation. Following fixation, cells were stained with a 2% AR solution at RT for 10 min then washed with deionized water and allowed to dry. Pictures were taken using Olympus Epson scanner (Los Angeles, CA, USA). In order to quantify bone nodule deposition, bone nodules were dissolved in 0.1 N sodium hydroxide and quantified using an Epoch microspectrophotometer (BioTek, Winooski, VT, USA) by absorbance at 548 nm. All experiments were performed with *n* = 3 human samples per anatomic depot, and in triplicate wells (biologic and technical triplicate).

### Adipogenic differentiation assays

CD146^+^ periosteal, adipose, and dermal pericytes or subpopulations based on CXCR4 expression of equal passage number were seeded in 24- or 48-well plates at a density of 2 × 10^5^ cells per mL and allowed to adhere overnight. In total, 24 h after seeding, basal medium was replaced with adipogenic differentiation medium, replenished every 3 days (Mesencult Adipogenic Differentiation medium, StemCell technologies Inc., Vancouver, BC). Oil red O staining was performed after 10–14 days differentiation. Cells were washed with PBS, and fixed with 4% paraformaldehyde for 15 min. After fixation, cells were washed with water and 500 μL Oil red O staining solution. Oil red O stock solution was prepared by dissolving 0.5 g of Oil red O in 100 mL isopropanol. Oil red O staining solution was prepared by dilution of stock solution with distilled water in a 3:2 ratio, followed by filtration. Oil red O staining was performed for 30 min at 37 °C. Following incubation, cells were washed with tap water followed by microscopy. All experiments were performed with *n* = 3 human samples per anatomic depot, and in triplicate wells (biologic and technical triplicate).

### Chondrogenic differentiation assays

CD146^+^ periosteal and adipose pericytes were suspended at a high density of 1 × 10^7^ cells per mL and seeded in 10 μL micromass culture in 6-well plates for 4 h incubation. After incubation, chondrogenic differentiation medium (MesenCultTM-ACF Chondrogenic Differentiation Basal Medium, STEMCELL Technologies, Cat# 05456) was gently added for chondrogenic differentiation over a 21 days period, with medium replenished every 3 days. After harvest, micromass cultures were cryosectioned at 12 μm thickness and stained with Alcian blue solution (1 g Alcian blue 8GX powder in the 100 mL 3% Acetic acid solution, pH 2.0). All experiments were performed with *n* = 3 human samples, and in triplicate wells (biologic and technical triplicate).

### Cord formation assays

CD146^+^ periosteal pericytes were labeled with PKH26 red fluorescent dye before use. Growth factor reduced Matrigel (BD Biosciences) was plated in 96-well culture plates and incubated at 37 °C to polymerize for 30 min. For the co-culture assay, HUVECs (Lonza, Cat # C2517A) (1.5 × 10^4^ cells/well) and CD146^+^ periosteal pericytes (500 cells/well) were seeded on the polymerized matrigel. The cells were co-cultured with media consisting of equal parts EGM2:DMEM (1:1 ratio) for 2 h. Tubule formation was observed at 2 h by microscopy (10×). All experiments were performed with *n* = 3 human samples, and in triplicate wells (biologic and technical triplicate).

### Proliferation

Proliferation assays were performed in 96-well plates (2 × 10^3^ cells/well) and assayed at 72 h using the CellTiter96® AQueous One Solution Cell Proliferation Assay kit (MTS, G358A; Promega, Madison, WI). Briefly, 20 μL of MTS solution was added to each well. After incubation for 1 h at 37 °C, the absorbance was measured at 490 nm using Epoch microspectrophotometer (BioTek).

### Migration

For the migration assays,^61^ 2.0 × 10^4^ CXCR4^−^ or CXCR4^+^ ASCs were resuspended in 100 μL of *α*-MEM medium without FBS and were placed in the upper well of 8-μm pore-size Transwell 24-insert plates (Corning, NY, USA). The migration assay was performed by adding to the bottom well 600 μL of α-MEM medium supplemented with 10% FBS or CXCL12 (50 ng/ml). After 4 h, cells on the bottom of the inserts were fixed with 100% methanol for 30 min and stained with 0.5% crystal violet (Sigma-Aldrich). Then cells that invaded into the lower surface were taken picture by using an 100× microscope (Leica). The stained chambers were eluted with 33% acetic acid solution and quantified the eluent by absorbance at 570 nm.

### Quantitative (q)RT-PCR

Specific gene expression among CD146^+^ periosteal, adipose, and dermal pericytes was assayed by qRT-PCR on days 0, 3, and 7 of osteogenic/adipogenic differentiation. Primers sequences are shown in Supplementary Table S3. Briefly, total RNA was extracted using TRIzol Reagent (Life technologies corporation). In total, 1 μg of total RNA from each sample was subjected to first-strand complementary deoxyribonucleic acid (cDNA) synthesis using the iScript™ cDNA Synthesis Kit (Bio-Rad) to a final volume of 20 μL. The reverse transcription reaction was performed at 25 °C for 5 min, followed by 46 °C for 20 min, and 95 °C for 1 min. For qRT-PCR, the reaction was performed using 2× SYBR green RT-PCR master mix and a QuantStudio 5 Real-Time PCR system instrument (Thermo Scientific, Waltham, MA). qRT-PCR was performed using 384-well optical plates at 95 °C for 10 min, followed by 40 cycles at 95 °C for 15 s, and at 60 °C for 60 s. The relative quantification of gene expression was performed using a Comparative CT method according to the manufacturer’s protocol and was normalized to the expression levels of housekeeping gene *ACTB* in each sample.

### Western blot

CD146^+^ periosteal, adipose, and dermal pericytes were lysed in RIPA buffer (Thermo Scientific) with protease inhibitor cocktail (Cell Signaling Technology, Danvers, MA, USA). Total proteins (20 μg) were separated by SDS–polyacrylamide gel electrophoresis and transferred to a nitrocellulose membrane, then blocked with 5% bovine serum albumin (BSA) and probed with rabbit antibodies against human CXCR4 and GAPDH at 4 °C overnight. Membranes were incubated with a horseradish-peroxidase (HRP)-conjugated secondary antibody and visualized with ChemiDoc XRS + System (Bio-rad). ImageJ (NIH, Bethesda, MD, USA) was used for densitometry.

### Microarray analysis

The transcriptome of CD146^+^ periosteal, adipose, and dermal pericytes was examined by expression microarray. Briefly, total RNA was extract from CD146^+^ pericytes by Trizol (Life technologies corporation). After purification and qualification, the RNA samples were sent to the JHMI Transcriptomics and Deep Sequencing Core (JHU, Baltimore, MD, USA) for analysis using an Affymetrix Clariom D microarray (Affymetrix, Santa Clara, CA, USA). Data analyses were performed using software packages including Partek Genomics Suite, Spotfire DecisionSite with Functional Genomics, and QIAGEN Ingenuity^®^ Pathway Analysis.

### Single-cell RNA (ScRNA) sequencing

Aligned scRNA-seq data were downloaded from Gene Expression Omnibus (GEO) under superseries accession number GSE128889. Briefly, subcutaneous fat was manually minced and digested with collagenase D and dispase II. Following centrifugation, supernatant containing mature adipocytes was aspirated, and the pellet, consisting of SVCs, was isolated and flow sorted with gating to isolate single cells away from debris, doublets, and dead cells, as well as gated against CD45 to exclude leukocytes. The library was generated using the 10X chromium controller and sequenced on an Ilumina HiSeq 2500. The resulting reads were aligned and demultiplexed using CellRanger. Cells were first filtered to have >500 and <6 000 detected genes, as well as <5% mitochondrial transcripts. Regression for nUMI, percent mitochondrial genes, and cell cycle, along with dimensionality reduction performed with the t-stochastic neighboring embedding method (tSNE), was performed using Seurat. tSNE, violin plots, and expression heatmaps were generated from a custom gene list using Seurat functions.

### Intramuscular implantation

Animals were housed and experiments were performed in accordance with institutional guidelines at the Johns Hopkins University, Maryland. All animal experiments were performed according to the approved protocol of the Animal Care and Use Committee (ACUC) at Johns Hopkins University (Approval No. MO16M302). A DBM Putty (DBX, courtesy of Musculoskeletal Transplant Foundation, Edison, NJ) was used for the ectopic bone formation assay in mice. Briefly, human CD146^+^ periosteal, adipose, or dermal pericytes were prepared at a density of 2.5 ×10^7^ cells per mL in PBS. For each implantation sample, 40 μL of cell suspension was mixed mechanically with 50 mg DBM Putty (1 million total cells in 40 μL PBS) and implanted intramuscularly into the thigh muscle pouch of 16-week-old male NOD/SCID mice (The Jackson Laboratory, Bar Harbor, Maine, USA). Briefly, animals were anesthetized by isoflurane inhalation and premedicated with buprenorphine. Incisions in the hindlimbs were made, and pockets were cut in the biceps femoris muscles by blunt dissection, parallel to the muscle fiber long axis. Dissection methods and the surgical manipulation of tissues were kept as constant as possible across animals. See Supplementary Table S4 for animals per experimental group. After implantation of the cell + scaffold composite, the fasciae overlying the muscle were sutured with a simple continuous pattern, and the skin was closed in a separate layer using 5-0 Vicryl (Ethicon, San Angelo, TX). In select experiments, AMD3100 (Sigma-Aldrich) or PBS vehicle control were administered according to prior reports^62^ (5 mg/kg, daily intraperitoneal injections). Surgeons were blinded to the experimental treatment groups during the procedure. In select studies, human CXCR4^−^ or CXCR4^+^ ASCs were implanted and analyzed in a similar fashion (implanted at a density of 7.5 × 10^7^ cells per mL in PBS, with 40 μL of cell suspension mixed mechanically with 50 mg DBM Putty).

### Microcomputed tomography imaging and analyses

Tissues were harvested and fixed in 4% PFA (paraformaldehyde) for 24 h, transferred to PBS, and scanned using a high-resolution microcomputed tomography (microCT) (SkyScan 1275; Bruker MicroCT N.V) at an image resolution of 15 μm, with the following settings: 1 mm aluminum filter, X-ray voltage of 65 kVP, anode current of 153 μA, exposure time of 160–218 ms, frame averaging of 6, and rotation step of 0.3 degrees. Three-dimensional images were then reconstructed from the 2D X-ray projections by implementing the Feldkamp algorithm using a commercial software package NRecon software (2.0.4.0 SkyScan). For the 3D morphometric analyses of images, CTVox and CTAn software (1.13 SkyScan) were used. Volumes of interest were shaped by polygons to cover the new bone. All analysis was performed in a blinded fashion.

### Histologic analysis

After radiographic imaging, samples were transferred to 14% EDTA (pH 7.6) for decalcification for 30 days. Samples were then embedded in optimal cutting temperature compound and sectioned in a coronal plane at 16 μm thickness. H&E staining were performed on serial sections. All images were examined with the microscopy of Leica DM6 B (Leica Microsystems Inc, Buffalo Grove, IL, USA). ALP staining was performed according to manufacturer’s instruction (Sigma-Aldrich).

### Whole mount immunohistochemistry

Human periosteum tissue was microdissected from a single patient sample (male donor, femoral periosteum). A 3 × 3 cm portion of periosteum was fixed in 4% PFA overnight, washed in tap water for 5 h, and then incubated with blocking buffer (0.1% Triton 100 and 1% BSA in PBS) overnight. Incubation with primary antibody (Rabbit polyclonal anti-CD146 antibody, Abcam, San Francisco, CA, USA; 1:50) was performed for 4 days with gentle rotation at 4 °C. A biotinylated goat anti-rabbit IgG antibody (1:200, Vector laboratories, BA-1000, Burlingame, CA, USA) was used as the secondary antibody, and ABC HRP kit (Vector Laboratories, PK-6100) and DAB solution were used for visualization (Vector Laboratories, SK-4100). The samples were examined with the microscopy of Motic smz-171 (Motic, Carlsbad, CA, USA).

### Indirect immunofluorescent staining

For additional immunofluorescent staining, either CD146^+^ periosteal pericytes in culture or cryosections (12–16 μm) of human periosteum, adipose, and intramuscular implants were used. All specimens were blocked with 5% serum in PBS for 1 h at 25 °C. Antigen retrieval was by trypsin enzymatic antigen retrieval solution (Cat# ab970; Abcam) for 5 min. Specimens were incubated with the following primary antibodies: anti-CD146 (Abcam, 1:50), anti-CD31 (Cell Signaling Technology, 1:320 or Abcam, 1:100), anti-Gli1 (Abcam, 1:100), anti-PDGFRα (Abcam, 1:200), anti-PDGFRβ (Abcam, 1:100), anti-OCN (Abcam, 1:200), and anti-CXCR4 (Abcam, 1:150). The Dylight 594 goat anti-rabbit IgG (H + L) polyclonal (Vector, 1:200), Goat anti-armenian hamster IgG (H+L) Alexa Fluor 647 (Abcam, 1:200) or Goat anti-mouse IgG (H + L) Alexa Fluor 488 or 647 (Abcam, 1:200) were used as secondary antibodies. Sections were counterstained with DAPI mounting medium (Cat# H-1500, Vector laboratories). All histological sections were examined with a Zeiss 780 confocal microscope (Zeiss, Thornwood, NY, USA) or microscopy of Leica DM6 B (Leica Microsystems Inc).

### Statistical analysis

Means and standard deviations were calculated from numerical data, as presented in the text, figures, and figure legends. In figures, bar graphs represent means, whereas error bars represent one standard deviation. The means of groups were compared using the Mann–Whitney *U* test when only two data sets were being compared and by the Kruskal–Wallis test with post hoc tests of Bonferroni to compare more than two groups. The statistical software SPSS for Windows Version 18.0 (SPSS) or GraphPad Prism (Version 7.0) were used for all statistical analyses. Statistical significance is indicated by *P < 0.05; **P < 0.01; and ***P < 0.001.

## Supplementary information

Supplemental Figures and Tables

Supplemental Figures and Tables

## Data Availability

Expression data that support the findings of this study have been deposited in Gene Expression Omnibus (GEO) with the accession codes GSE125545.
