# Supplementary material for: Comparison of skeletal and soft tissue pericytes identifies CXCR4+ bone forming mural cells in human tissues
Source: Bone Res. 2020 May 22;8:22. doi: 10.1038/s41413-020-0097-0 (PMC7244476; doi:10.1038/s41413-020-0097-0)
Supplement: Supplementary file 1 — Supplemental Figures and Tables [file 41413_2020_97_MOESM1_ESM.docx]

**Supplementary Data**

**Supplementary Figures and Figure Legends**

**Supplementary Figure S1**

**
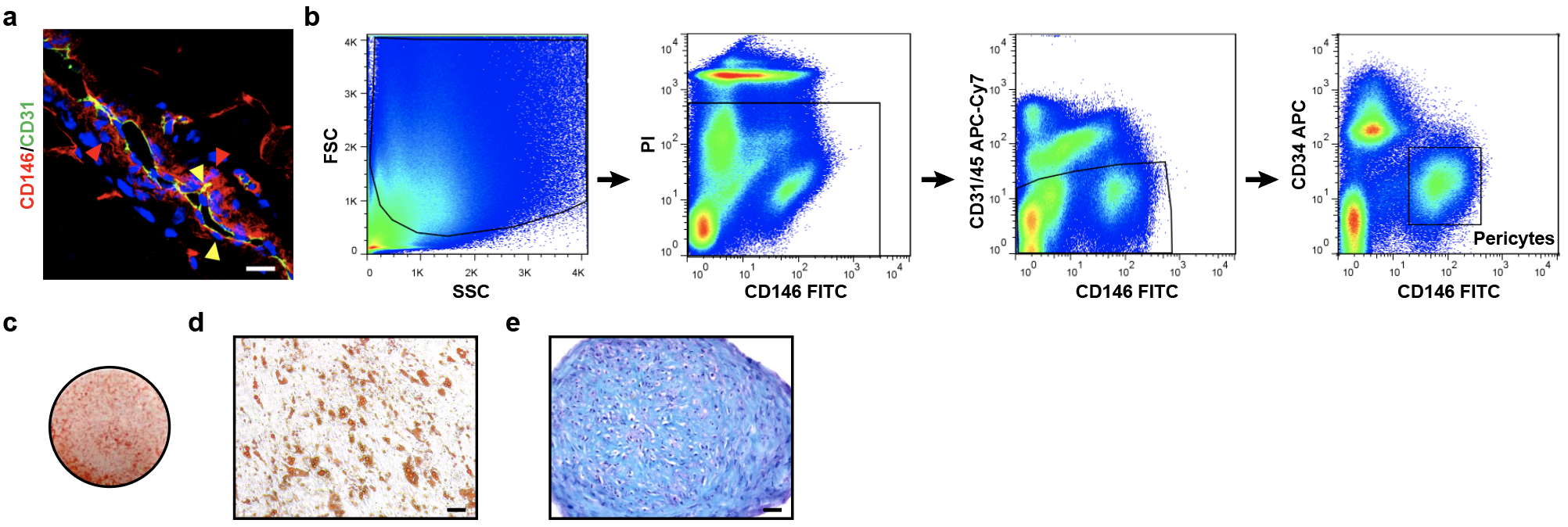
**

**Supplementary Figure S1.** Identification, isolation by fluorescent activated cell sorting (FACS) and multilineage differentiation of CD146^+^ pericytes from human adipose tissue. **(a)** Immunofluorescent staining of pericytes within adipose tissue blood vessels. Merged image, highlighting CD146^+^CD31^-^ pericyte (red arrowhead), and CD31^+^CD146^+^ endothelium (yellow arrowheads). White scale bar: 20 μm. **(b)** From left to right: pericytes are derived by size distribution, followed by exclusion of PI^+^ cells, followed by exclusion of CD31 or CD45 expressing cells. Finally, microvascular pericytes are derived as a CD146^+^CD34^-^ cell population. **(c)** Osteogenic differentiation, as assessed by alizarin red staining at 10 d of differentiation. **(d)** Adipogenic differentiation, as assessed by oil red O staining at 10 d of differentiation. **(e)** Chondrogenic differentiation, as assessed by alcian blue staining at 21 d of differentiation in high density micromass culture. Black scale bar: 50 μm.

**Supplementary Figure S2**

**
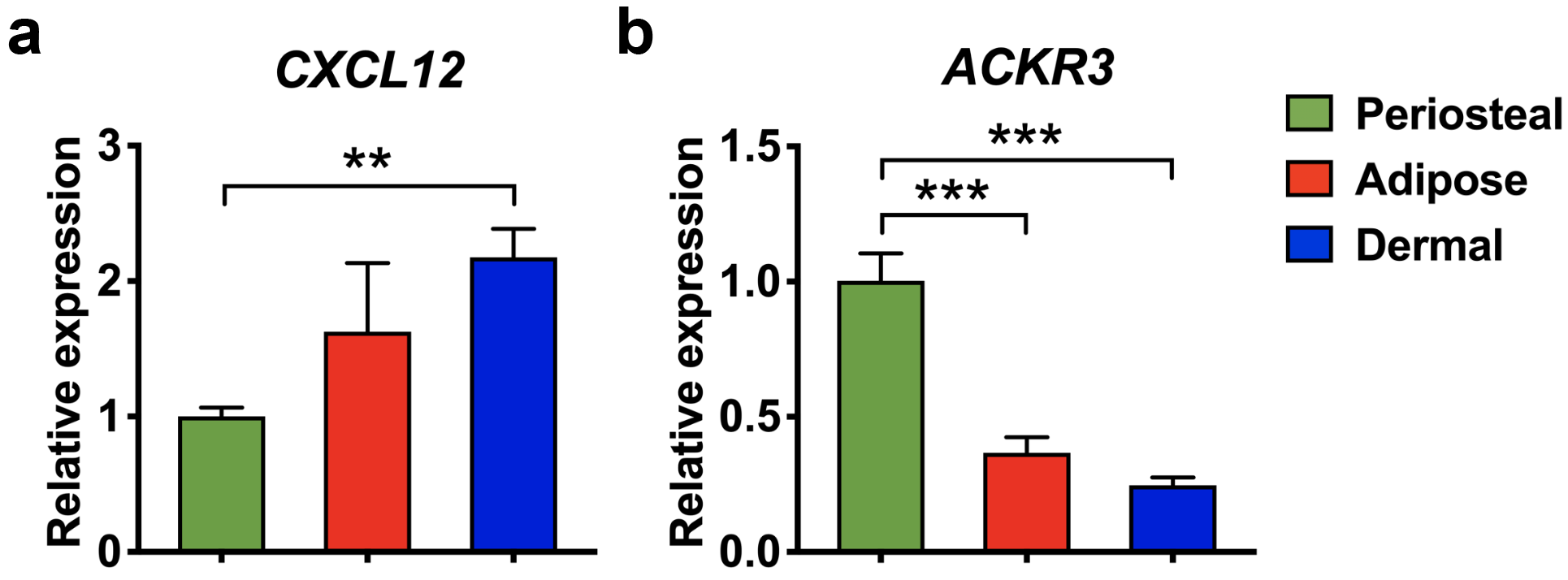
**

**Supplementary Figure S2.** Representative qRT-PCR analyses of **(a)** *CXCL12* and **(b)** *ACKR3 (CXCR7)* expression among CD146^+^ periosteal, adipose, and dermal pericytes. ***P*<0.01; ****P*<0.001.

**Supplementary Figure S3**


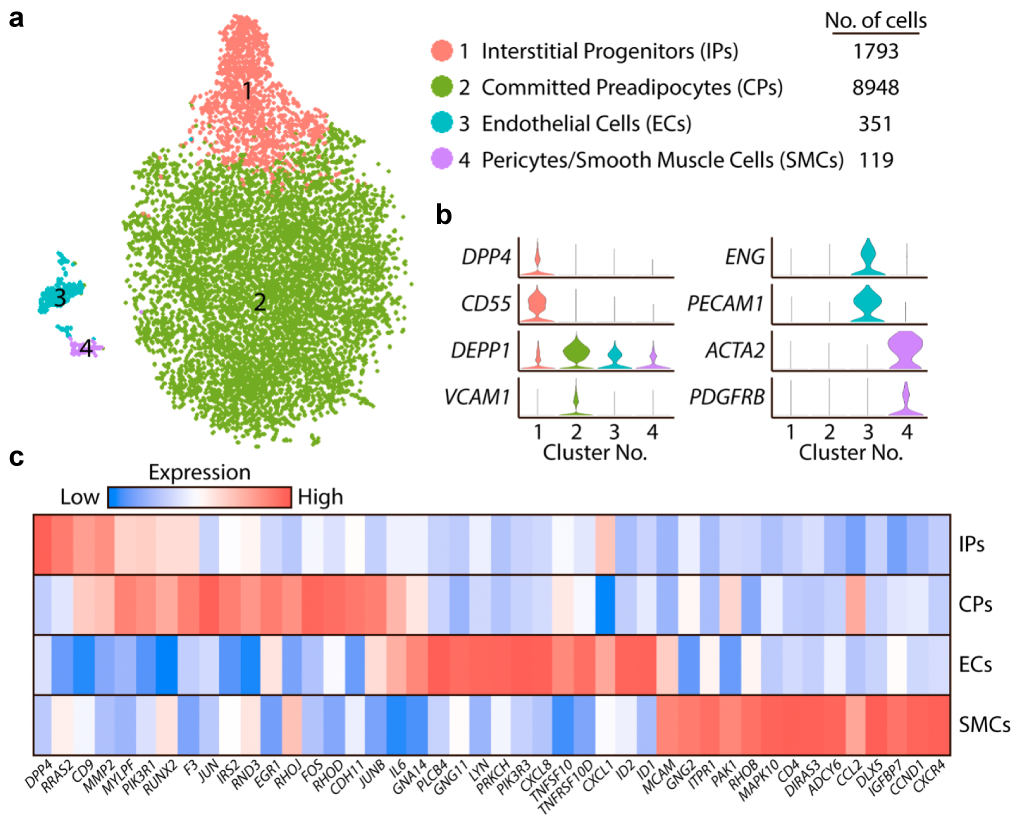


**Supplementary Figure S3.** CXCR4 signaling pathway analysis from single-cell RNA sequencing of human adipose tissue. **(a)** Re-analysis of single-cell RNA sequencing of human subcutaneous fat identified four unique cell populations, including interstitial progenitors (IPs, cluster 1), committed pre-adipocytes (CPs, cluster 2), endothelial cells (ECs, cluster 3), and smooth muscle cell / pericytes (SMCs, cluster 4). **(b)** Violin plots of known marker genes identify cell clusters as IPs (*DPP4*, *CD55*), CPs (*DEPP1*, *VCAM1*), ECs (*ENG*, *PECAM1*), and SMC/pericytes (*ACTA2*, *PDGFRB*). **(c)** SMC/pericytes show abundant expression of genes involved in the CXCR4 signaling pathway, including *CXCR4* itself.

**Supplementary Figure S4**

**
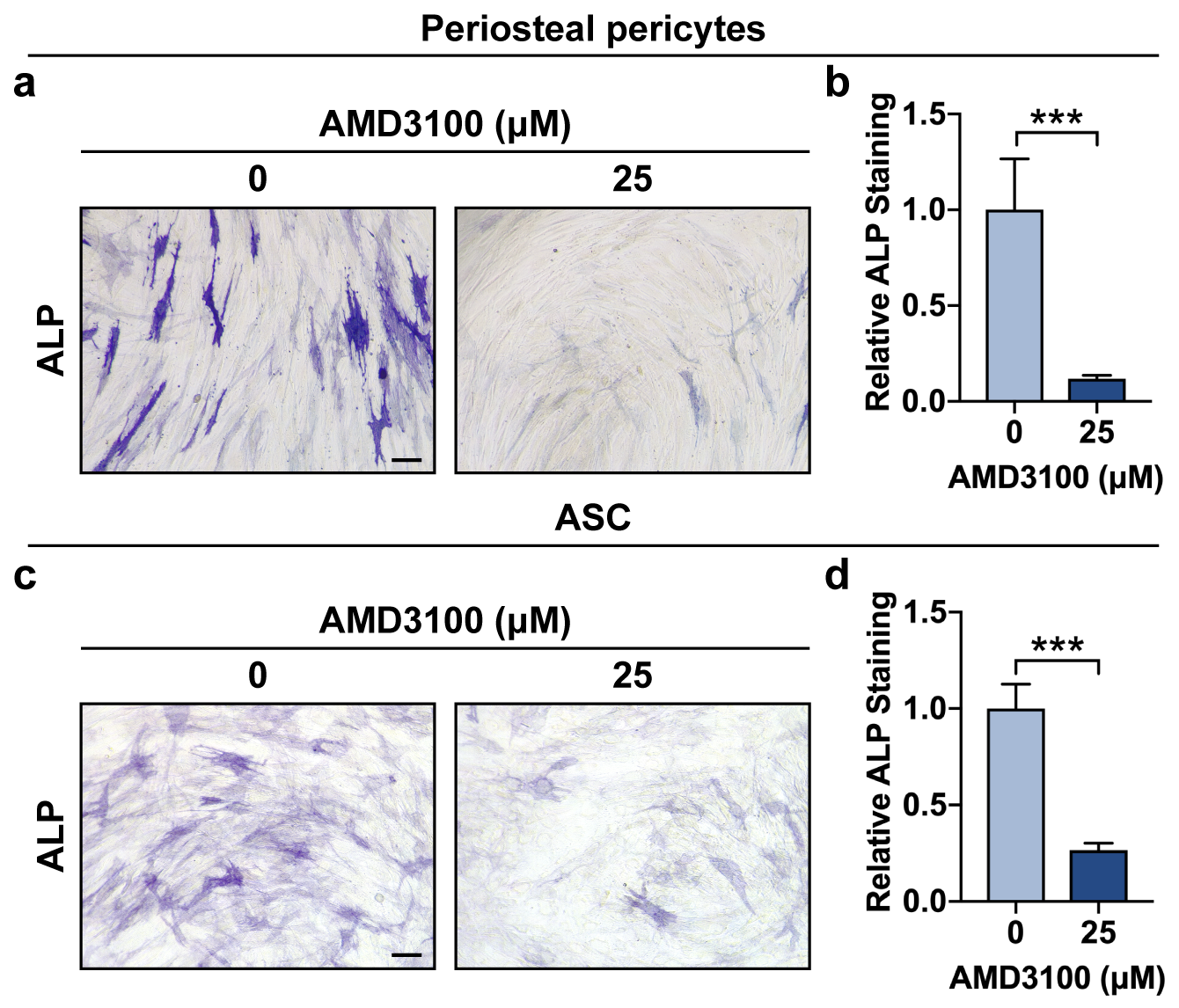
**

**Supplementary Figure S4.** CXCR4 inhibition impairs osteogenic differentiation of either periosteal pericytes or adipose tissue stromal cells (ASCs). **(a,b)** Alkaline phosphatase staining **(a)** and quantification **(b)** at d3 of osteogenic differentiation among periosteal pericytes treated with or without AMD3100. **(c,d)** Alkaline phosphatase staining **(c)** and quantification **(d)** at d3 of osteogenic differentiation among ASCs treated with or without AMD3100. Black scale bar: 100 μm. ****P*<0.001.

**Supplementary Figure S5**

**
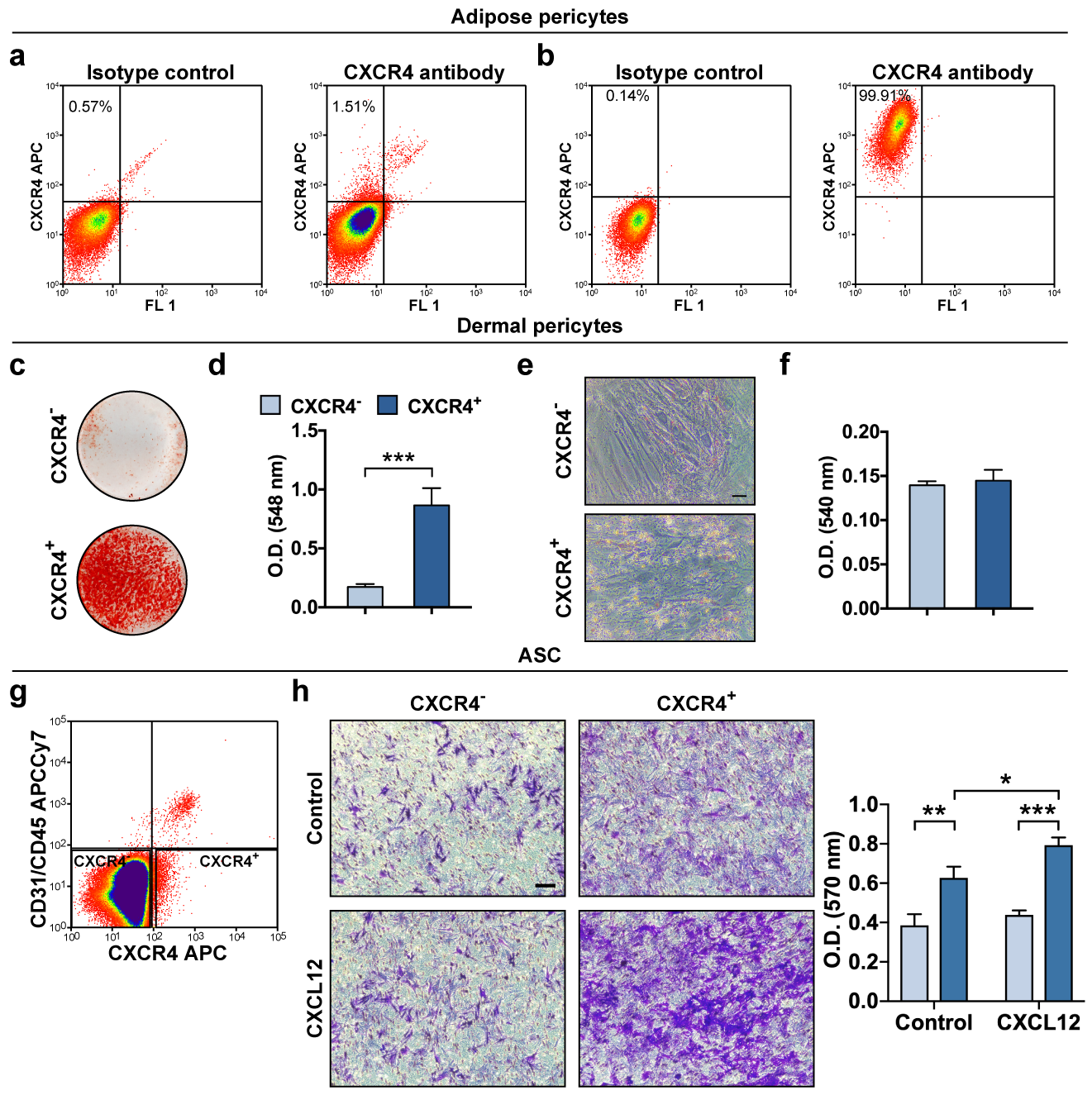
**

**Supplementary Figure S5.** Isolation and characterization of CXCR4^-^ and CXCR4^+^ cells from human soft tissue sources. **(a)** Isolation of CXCR4^-/+^ subsets was performed from CD146^+^ human adipose tissue pericytes. Isotype control staining shown. **(b)** FACS analysis of intracellular CXCR4 in CD146^+^ human adipose tissue pericytes. **(c,d)** Osteogenic differentiation of CXCR4^-^ and CXCR4^+^ dermal pericytes, including **(c)** alizarin red staining and **(d)** photometric quantification at d 7 of osteogenic differentiation. **(e,f)** Adipogenic differentiation of CXCR4^-^ and CXCR4^+^ dermal pericytes, including **(e)** oil red O staining and **(f)** photometric quantification at d 14 of adipogenic differentiation. **(g)** Isolation of CXCR4^-/+^ subsets was performed from culture-derived total adipose-derived stromal cells (ASCs). Differential proliferation and migration among CXCR4^-^ and CXCR4^+^ ASC. **(h)** CXCR4^-^ or CXCR4^+^ ASC migration assessed by transwell assay at 4 h with or without CXCL12 (50 ng/mL) treatment. Black scale bar: 100 μm. **P*<0.05; ***P*<0.01; ****P*<0.001.

**Supplementary Figure S6**


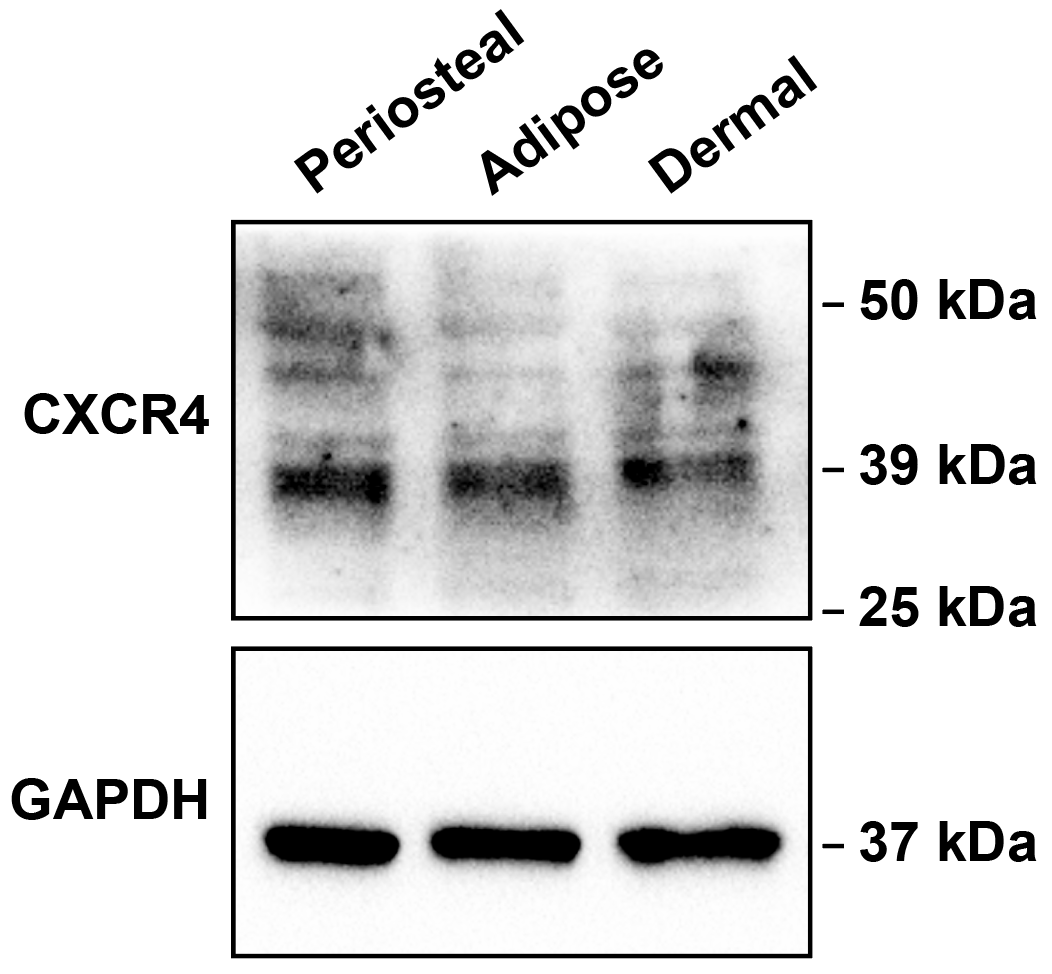


**Supplementary Figure S6.** Uncropped versions of representative western blot images from Fig. 4h.

**Supplementary Tables**

**Supplementary Table S1.** Frequency of CD146^+^CD31^-^CD45^-^ pericytes by anatomic depot of origin. Percentage reflects numbers of CD146^+^CD31^-^CD45^-^ events as a function of total FACS events.

| **Patient No.** | **Tissue type** | **Sample source** | **CD146^+^CD31^-^CD45^-^(%)** |
| --- | --- | --- | --- |
| 1 | Periosteum | Tibia | 1.3 |
| 2 | Periosteum | Tibia | 6.5 |
| 3 | Periosteum | Metatarsus | 6.8 |
| 4 | Subcutaneous adipose | Lipoaspirate | 9.0 |
| 5 | Subcutaneous adipose | Lipoaspirate | 1.3 |
| 6 | Subcutaneous adipose | Lipoaspirate | 5.6 |
| 7 | Dermis | Abdominoplasty skin | 4.5 |
| 8 | Dermis | Abdominoplasty skin | 14.0 |
| 9 | Dermis | Abdominoplasty skin | 16.9 |

**Supplementary Table S2.** Antibodies used.

| **Antibody** | **Company** | **Catalog #** | **Use** |
| --- | --- | --- | --- |
| Mouse anti-Human CD31 | BD Pharmingen | 563653 | FACS / F |
| Mouse anti-Human CD34 | BD Pharmingen | 555824 | FACS |
| Mouse anti-Human CD44 | BD Pharmingen | 561289 | F |
| Mouse anti-Human CD45 | BD Pharmingen | 557833 | FACS / F |
| Mouse anti-Human CD73 | BD Pharmingen | 561014 | F |
| Mouse anti-Human CD90 | BD Pharmingen | 555595 | F |
| Mouse anti-Human CD105 | BD Pharmingen | 562380 | F |
| Mouse anti-Human CD146 | Bio-Rad | MCA2141F | FACS |
| Mouse anti-Human CXCR4 | BD Pharmingen | 555976 | FACS |
| Mouse anti-CD31 | Cell Signaling Technology | 3528S | IF |
| Armenian hamster anti-CD31 | Abcam | ab119341 | IF |
| Rabbit anti-CD146 | Abcam | ab75769 | ICC / IF / IHC |
| Mouse anti-CD146 | Abcam | ab24577 | IF |
| Rabbit anti-human CXCR4 | Abcam | ab124824 | IF / WB |
| Rabbit anti-human GAPDH | Cell Signaling Technology | 2118 | WB |
| Rabbit anti-Gli1 | Abcam | ab49314 | ICC |
| Rabbit anti-Osteocalcin | Abcam | ab93876 | IF |
| Rabbit anti-PDGFRA | Abcam | ab15501 | ICC |
| Rabbit anti-PDGFRB | Abcam | ab32570 | ICC |
| Goat anti-rabbit IgG(H+L) | Vector Laboratories | DI1594 | ICC / IF |
| Goat anti-mouse IgG(H+L) | Abcam | ab150119 | ICC / IF |
| Goat anti-armenian hamster IgG(H+L) | Abcam | ab173004 | IF |
| Anti-rabbit IgG, HRP-linked Antibody | Cell Signaling Technology | 7074 | WB |
| F: Flow cytometry; FACS: Fluorescent activated cell sorting; ICC: Immunocytochemistry; IF: Immunofluorescent staining; IHC: Immunohistochemistry; WB: Western blot | | | |

**Supplementary Table S3.** Primers used.

| Genes (human) | Forward | Reverse |
| --- | --- | --- |
| *ACKR3* | 5’-TCTGCATCTCTTCGACTACTCA-3’ | 5’-GTAGAGCAGGACGCTTTTGTT-3’ |
| *ACTB* | 5’-CTGGAACGGTGAAGGTGACA-3’ | 5’-AAGGGACTTCCTGTAACAATGCA-3’ |
| *ALP* | 5’-ACCACCACGAGAGTGAACCA-3’ | 5’-CGTTGTCTGAGTACCAGTCCC-5’ |
| *αSMA* | 5’-AAAAGACAGCTACGTGGGTGA-3’ | 5’-GCCATGTTCTATCGGGTACTTC-3’ |
| *CD146* | 5’-AGCTCCGCGTCTACAAAGC-3’ | 5’-CTACACAGGTAGCGACCTCC-3’ |
| *CEBPα* | 5’-TGGACAAGAACAGCAACGAGTA-3’ | 5’-ATTGTCACTGGTCAGCTCCAG-3’ |
| *COL1A1* | 5’-GAGGGCCAAGACGAAGACATC-3’ | 5’-CAGATCACGTCATCGCACAAC-3’ |
| *CXCL12* | 5’-ATTCTCAACACTCCAAACTGTGC-3’ | 5’-ACTTTAGCTTCGGGTCAATGC-3’ |
| *CXCR4* | 5’-GGGCAATGGATTGGTCATCCT-3’ | 5’-TGCAGCCTGTACTTGTCCG-3’ |
| *NG2* | 5’-GCCACGTTGTCAGTCGATG-3’ | 5’-CCCATAGGGGACCTCTAGGG-3’ |
| *OCN* | 5’-CACTCCTCGCCCTATTGGC-3’ | 5’-CCCTCCTGCTTGGACACAAAG-3’ |
| *OSX* | 5’-CCTCTGCGGGACTCAACAAC-3’ | 5’-AGCCCATTAGTGCTTGTAAAGG-3’ |
| *PPARγ* | 5’-GACAGGAAAGACAACAGACAAATC-3’ | 5’-GGGGTGATGTGTTTGAACTTG-3’ |
| *RUNX2* | 5’-TGGTTACTGTCATGGCGGGTA-3’ | 5’-TCTCAGATCGTTGAACCTTGCTA-3’ |

**Supplementary Table S4.** Animal allocation.

| **Cell group** | **Scaffold** | **Cell #** | **Animal #** |
| --- | --- | --- | --- |
| Scaffold only | DBX Putty (50 mg) | 0 | 3 |
| Periosteal pericytes |  | 1.0 × 10^6^ | 3 |
| Adipose pericytes |  | 1.0 × 10^6^ | 3 |
| Dermal pericytes |  | 1.0 × 10^6^ | 3 |

| **Cell group** | **Scaffold** | **Treatment** | **Cell #** | **Animal #** |
| --- | --- | --- | --- | --- |
| Periosteal pericytes | DBX Putty (50 mg) | Control | 1.0 × 10^6^ | 3 |
|  |  | AMD3100 |  | 3 |
| Adipose pericytes |  | Control | 1.0 × 10^6^ | 3 |
|  |  | AMD3100 |  | 3 |

| **Cell group** | **Scaffold** | **Cell #** | **Animal #** |
| --- | --- | --- | --- |
| CXCR4^-^ ASC | DBX Putty (50 mg) | 3.0 × 10^6^ | 3 |
| CXCR4^+^ ASC |  | 3.0 × 10^6^ | 3 |

**Supplementary Table S5.** Relative expression of pericyte markers in periosteal, adipose and dermal pericytes.

| ***Gene Symbol (NCBI Jun-18)*** | ***Periosteal_1Way vs. Adipose Log2(FC)*** | ***Periosteal_1Way vs. Adipose (p-val)*** | ***Periosteal_1Way vs. Dermal Log2(FC)*** | ***Periosteal_1Way vs. Dermal (p-val)*** |
| --- | --- | --- | --- | --- |
| *THY1* | -0.86585 | 0.294603 | -0.935241 | 0.261201 |
| *NT5E* | 0.272051 | 0.553682 | 0.334313 | 0.470168 |
| *ENG* | 0.173687 | 0.487409 | 0.660639 | 0.0306151 |
| *CD44* | -0.245921 | 0.186268 | -0.0410938 | 0.811417 |
| *ACTA2* | 0.461642 | 0.697629 | 0.489966 | 0.680329 |
| *CSPG4* | 0.201341 | 0.77879 | -0.378358 | 0.60079 |
| *ANPEP* | -0.192041 | 0.760033 | -0.0350513 | 0.955363 |
| *ABCC9* | -1.61272 | 0.328563 | -0.705363 | 0.658292 |
| *DLK1* | -0.309954 | 0.109182 | -0.165868 | 0.353265 |
| *RGS5* | 0.370364 | 0.679547 | 0.151946 | 0.864579 |
| *KCNJ8* | -0.8901 | 0.132227 | -0.298251 | 0.58078 |
| *CD248* | -0.961579 | 0.053871 | -0.491334 | 0.267432 |
| *GLI1* | 0.0748895 | 0.703296 | 0.0592895 | 0.762453 |
| *MCAM* | 0.953843 | 0.164806 | -0.283748 | 0.654602 |
| *LEPR* | 0.994598 | 0.123627 | 1.15757 | 0.0823373 |
| *TBX18* | -0.130813 | 0.627709 | -0.55122 | 0.0748401 |
| *PDGFRA* | -0.0224145 | 0.964381 | 0.738259 | 0.176105 |
| *PDGFRB* | -0.0869417 | 0.916489 | 0.276081 | 0.740264 |

**Supplementary Table S6.** Relative expression of common angiogenesis cytokines in periosteal, adipose and dermal pericytes.

| ***Gene Symbol (NCBI Jun-18)*** | ***Periosteal_1Way vs. Adipose Log2(FC)*** | ***Periosteal_1Way vs. Adipose (p-val)*** | ***Periosteal_1Way vs. Dermal Log2(FC)*** | ***Periosteal_1Way vs. Dermal (p-val)*** |
| --- | --- | --- | --- | --- |
| *VEGFA* | -0.347435 | 0.353692 | 0.21114 | 0.56377 |
| *VEGFB* | -0.0987606 | 0.717949 | -0.166626 | 0.546469 |
| *VEGFC* | 0.20976 | 0.748012 | -0.0669314 | 0.918014 |
| *VEGFD* | -0.197408 | 0.548508 | 0.170035 | 0.60382 |
| *ANGPT1* | 0.533114 | 0.426089 | 0.517594 | 0.438965 |
| *ANGPT2* | -1.54173 | 0.110693 | -0.0260539 | 0.975816 |
| *TGFB1* | -0.344493 | 0.17419 | 0.551564 | 0.0486101 |
| *CCL2* | 1.55033 | 0.0623926 | 1.86282 | 0.0335013 |
| *CCL3* | 0.189754 | 0.275954 | 0.150128 | 0.379673 |
| *CXCL16* | -0.668861 | 0.305643 | 0.0921036 | 0.882517 |
| *HGF* | -0.0661488 | 0.952185 | 2.56971 | 0.0512684 |
| *FGF4* | 0.0691616 | 0.466944 | -0.0554117 | 0.55674 |
| *FGF7* | -0.863451 | 0.644804 | -0.922845 | 0.622685 |
| *SERPINE1* | 0.266965 | 0.649973 | 0.607085 | 0.319332 |
| *SERPINF1* | -0.526099 | 0.544903 | 0.491345 | 0.570999 |
| *ADAMTS1* | 0.100496 | 0.82508 | -0.288682 | 0.531828 |
| *INHBA* | 0.38688 | 0.73517 | 0.512848 | 0.655102 |
| *PIGF* | -0.0974382 | 0.513209 | -0.147025 | 0.334882 |
| *EDN1* | 0.599615 | 0.584247 | 0.104845 | 0.922782 |
| *THBS1* | -0.206194 | 0.536516 | -0.174709 | 0.598753 |
| *THBS2* | 1.39252 | 0.399246 | 2.45124 | 0.161375 |
| *PRL* | 0.0858279 | 0.43498 | 0.111645 | 0.318372 |
| *TIMP1* | -0.0198167 | 0.927682 | 0.26122 | 0.258686 |
| *TIMP4* | -0.775925 | 0.124526 | 0.498388 | 0.295253 |
| *MMP8* | 0.490809 | 0.0107382 | 0.302864 | 0.0653717 |
| *MMP9* | -0.225617 | 0.193336 | 0.0840522 | 0.604963 |
| *IL1B* | -1.61615 | 0.052425 | 0.260424 | 0.710931 |
| *COL18A1* | -0.398107 | 0.530561 | -0.0891089 | 0.886492 |
| *IGBP1* | 0.00420125 | 0.971111 | -0.179525 | 0.157833 |

**Supplementary Table S7.** Most significantly upregulated (periosteal vs adipose) by Ingenuity pathway analysis.

| **Pathway** | **Z score** | **Negative Log10(p-value)** |
| --- | --- | --- |
| CXCR4 Signaling | 2.887 | 0.596 |
| Th2 Pathway | 2.828 | 0.79 |
| Huntington's Disease Signaling | 2.828 | 0 |
| PAK Signaling | 2.714 | 1.18 |
| GPCR-Mediated Nutrient Sensing in Enteroendocrine Cells | 2.646 | 0.267 |
| Neuropathic Pain Signaling In Dorsal Horn Neurons | 2.646 | 0 |
| Integrin Signaling | 2.53 | 0 |
| BMP signaling pathway | 2.449 | 0.64 |
| NF-κB Activation by Viruses | 2.449 | 0.29 |
| G Beta Gamma Signaling | 2.449 | 0 |
| Cell Cycle: G2/M DNA Damage Checkpoint Regulation | 2.333 | 3.06 |
| Sumoylation Pathway | 2.333 | 0.846 |
| Renin-Angiotensin Signaling | 2.333 | 0.405 |
| Thrombin Signaling | 2.333 | 0.392 |
| Tec Kinase Signaling | 2.333 | 0.378 |
| Role of NFAT in Cardiac Hypertrophy | 2.309 | 0 |
| Pancreatic Adenocarcinoma Signaling | 2.236 | 1.15 |
| Paxillin Signaling | 2.236 | 0.372 |
| Melatonin Signaling | 2.236 | 0.354 |
| fMLP Signaling in Neutrophils | 2.236 | 0 |
| Adrenomedullin signaling pathway | 2 | 0.998 |
| Calcium-induced T Lymphocyte Apoptosis | 2 | 0.347 |
| Activation of IRF by Cytosolic Pattern Recognition Receptors | 2 | 0.275 |
| 14-3-3-mediated Signaling | 2 | 0 |
| LPS-stimulated MAPK Signaling | 2 | 0 |
| GP6 Signaling Pathway | 1.941 | 1.21 |
| Role of Pattern Recognition Receptors in Recognition of Bacteria and Viruses | 1.897 | 1.79 |
| HMGB1 Signaling | 1.897 | 0.632 |
| Gα12/13 Signaling | 1.897 | 0.438 |
| Coagulation System | 1.89 | 2.29 |
| Agrin Interactions at Neuromuscular Junction | 1.89 | 1.11 |
| Cdc42 Signaling | 1.89 | 0.458 |
| CREB Signaling in Neurons | 1.89 | 0 |
| Cardiac Hypertrophy Signaling | 1.807 | 0.303 |
| Gαq Signaling | 1.732 | 0.554 |

**Supplementary Table S8.** Most significantly downregulated (periosteal vs adipose) by Ingenuity pathway analysis.

| **Pathway** | **Z score** | **Negative Log10(p-value)** |
| --- | --- | --- |
| Cyclins and Cell Cycle Regulation | -3.464 | 2.9 |
| Aryl Hydrocarbon Receptor Signaling | -2.673 | 3.2 |
| Heparan Sulfate Biosynthesis | -2.646 | 0.993 |
| Heparan Sulfate Biosynthesis (Late Stages) | -2.449 | 0.848 |
| Mitotic Roles of Polo-Like Kinase | -2.309 | 5.99 |
| Dermatan Sulfate Biosynthesis (Late Stages) | -2.236 | 0.929 |
| Triacylglycerol Biosynthesis | -2.236 | 0.866 |
| Neuroprotective Role of THOP1 in Alzheimer's Disease | -2.236 | 0 |
| Estrogen-mediated S-phase Entry | -2.121 | 3.9 |
| γ-linolenate Biosynthesis II (Animals) | -2 | 1.73 |
| Cell Cycle Regulation by BTG Family Proteins | -2 | 1.11 |
| Role of BRCA1 in DNA Damage Response | -1.941 | 5.17 |
| Salvage Pathways of Pyrimidine Ribonucleotides | -1.941 | 2.27 |
| LXR/RXR Activation | -1.941 | 1.5 |
| Complement System | -1.89 | 2.86 |
| Chondroitin Sulfate Biosynthesis | -1.89 | 1.35 |
| Dermatan Sulfate Biosynthesis | -1.89 | 1.27 |
| Role of IL-17F in Allergic Inflammatory Airway Diseases | -1.633 | 1.4 |
| Chondroitin Sulfate Biosynthesis (Late Stages) | -1.633 | 1.23 |
| RhoGDI Signaling | -1.604 | 1.1 |
| Pyridoxal 5'-phosphate Salvage Pathway | -1.508 | 2.74 |
| p38 MAPK Signaling | -1.414 | 0.381 |
| B Cell Receptor Signaling | -1.134 | 0 |
| Oncostatin M Signaling | -1 | 0.998 |
| Ethanol Degradation II | -1 | 0.907 |
| Pyrimidine Ribonucleotides De Novo Biosynthesis | -1 | 0.534 |
| Acute Phase Response Signaling | -0.816 | 0.407 |
| Production of Nitric Oxide and Reactive Oxygen Species in Macrophages | -0.775 | 0.665 |
| PPAR Signaling | -0.707 | 0.807 |
| STAT3 Pathway | -0.707 | 0.508 |
| ERK/MAPK Signaling | -0.632 | 0 |
| Noradrenaline and Adrenaline Degradation | -0.447 | 1.29 |
| Phospholipases | -0.447 | 0.534 |
| Antioxidant Action of Vitamin C | -0.447 | 0.336 |
| NGF Signaling | -0.447 | 0 |

**Supplementary Table S9.** Most significantly upregulated (periosteal vs dermal) by Ingenuity pathway analysis.

| **Pathway** | **Z score** | **Negative Log10(p-value)** |
| --- | --- | --- |
| TREM1 Signaling | 3 | 2.1 |
| ILK Signaling | 2.84 | 1.47 |
| IL-8 Signaling | 2.828 | 1.9 |
| Protein Kinase A Signaling | 2.746 | 3.06 |
| Regulation of Actin-based Motility by Rho | 2.333 | 1.55 |
| PAK Signaling | 2.138 | 3.09 |
| Dendritic Cell Maturation | 2.138 | 1.24 |
| GP6 Signaling Pathway | 2 | 3.1 |
| Inflammasome pathway | 2 | 1.8 |
| Interferon Signaling | 2 | 1.49 |
| MIF-mediated Glucocorticoid Regulation | 2 | 1.09 |
| Calcium-induced T Lymphocyte Apoptosis | 2 | 0.528 |
| RhoA Signaling | 1.941 | 2.12 |
| BMP signaling pathway | 1.897 | 3.79 |
| Corticotropin Releasing Hormone Signaling | 1.897 | 1.79 |
| Cdc42 Signaling | 1.897 | 1.71 |
| Death Receptor Signaling | 1.89 | 0.75 |
| HMGB1 Signaling | 1.807 | 2.86 |
| p38 MAPK Signaling | 1.732 | 1.93 |
| Signaling by Rho Family GTPases | 1.706 | 3.12 |
| Colorectal Cancer Metastasis Signaling | 1.706 | 2.16 |
| Th1 Pathway | 1.667 | 1.03 |
| MIF Regulation of Innate Immunity | 1.633 | 1.81 |
| tRNA Splicing | 1.633 | 1.76 |
| Activation of IRF by Cytosolic Pattern Recognition Receptors | 1.633 | 1.08 |
| Phospholipase C Signaling | 1.604 | 0.996 |
| Thrombin Signaling | 1.508 | 2.11 |
| GPCR-Mediated Nutrient Sensing in Enteroendocrine Cells | 1.508 | 1.63 |
| Actin Cytoskeleton Signaling | 1.508 | 1.19 |
| Integrin Signaling | 1.5 | 1.45 |
| Gα12/13 Signaling | 1.414 | 3.69 |
| IL-1 Signaling | 1.414 | 1.81 |
| cAMP-mediated signaling | 1.342 | 2.06 |
| Gαq Signaling | 1.291 | 1.88 |
| GNRH Signaling | 1.291 | 2.71 |
| Ephrin Receptor Signaling | 1.134 | 0.761 |
| CXCR4 Signaling | 1 | 2.31 |

**Supplementary Table S10.** Most significantly downregulated (periosteal vs dermal) by Ingenuity pathway analysis.

| **Pathway** | **Z score** | **Negative Log10(p-value)** |
| --- | --- | --- |
| Neurotrophin/TRK Signaling | -2.449 | 0.914 |
| LXR/RXR Activation | -2.309 | 1.82 |
| ErbB2-ErbB3 Signaling | -2 | 0.505 |
| RhoGDI Signaling | -1.807 | 2.52 |
| Superpathway of Inositol Phosphate Compounds | -1.732 | 0.325 |
| PPAR Signaling | -1.667 | 1.6 |
| GDNF Family Ligand-Receptor Interactions | -1.633 | 0.934 |
| Renal Cell Carcinoma Signaling | -1.633 | 0.855 |
| IL-3 Signaling | -1.633 | 0.541 |
| 3-phosphoinositide Biosynthesis | -1.508 | 0.419 |
| CDK5 Signaling | -1.414 | 1.45 |
| Role of NFAT in Regulation of the Immune Response | -1.414 | 0.552 |
| 3-phosphoinositide Degradation | -1.414 | 0.312 |
| D-myo-inositol-5-phosphate Metabolism | -1.414 | 0.286 |
| Prolactin Signaling | -1.342 | 0.541 |
| Ceramide Signaling | -1.342 | 0.268 |
| Acute Myeloid Leukemia Signaling | -1.342 | 0.268 |
| Telomerase Signaling | -1.342 | 0 |
| Insulin Receptor Signaling | -1.342 | 0 |
| B Cell Receptor Signaling | -1.265 | 0.341 |
| Antioxidant Action of Vitamin C | -1.134 | 1.13 |
| D-myo-inositol (1,4,5,6)-Tetrakisphosphate Biosynthesis | -1.134 | 0.273 |
| D-myo-inositol (3,4,5,6)-tetrakisphosphate Biosynthesis | -1.134 | 0.273 |
| Glutathione-mediated Detoxification | -1 | 1.59 |
| IGF-1 Signaling | -1 | 1.31 |
| Bladder Cancer Signaling | -1 | 1.03 |
| UVA-Induced MAPK Signaling | -1 | 1.02 |
| FcγRIIB Signaling in B Lymphocytes | -1 | 0.874 |
| Leptin Signaling in Obesity | -1 | 0.874 |
| Thrombopoietin Signaling | -1 | 0.32 |
| ErbB4 Signaling | -1 | 0.274 |
| GM-CSF Signaling | -1 | 0.249 |
| Melanocyte Development and Pigmentation Signaling | -1 | 0 |
| PKCθ Signaling in T Lymphocytes | -1 | 0 |
| Salvage Pathways of Pyrimidine Ribonucleotides | -1 | 0 |
| JAK/Stat Signaling | -1 | 0 |
